# A Systematic Review of Culture-Based Methods for Monitoring Antibiotic-Resistant *Acinetobacter*, *Aeromonas*, and *Pseudomonas* as Environmentally Relevant Pathogens in Wastewater and Surface Water

**DOI:** 10.1007/s40572-023-00393-9

**Published:** 2023-02-23

**Authors:** Erin G. Milligan, Jeanette Calarco, Benjamin C. Davis, Ishi M. Keenum, Krista Liguori, Amy Pruden, Valerie J. Harwood

**Affiliations:** 1 Department of Civil and Environmental Engineering, Virginia Tech, Blacksburg, VA 24061 USA; 2 Center for Emerging, Zoonotic, and Arthropod-Borne Pathogens, Virginia Polytechnic Institute and State University, Blacksburg, VA 24061 USA; 3 Department of Integrative Biology, University of South Florida, Tampa, FL 33620 USA

**Keywords:** Antibiotic resistance, Environmental monitoring, Wastewater, Surface water, Environmental pathogens

## Abstract

**Purpose of Review:**

Mounting evidence indicates that habitats such as wastewater and environmental waters are pathways for the spread of antibiotic-resistant bacteria (ARB) and mobile antibiotic resistance genes (ARGs). We identified antibiotic-resistant members of the genera *Acinetobacter*, *Aeromonas*, and *Pseudomonas* as key opportunistic pathogens that grow or persist in built (e.g., wastewater) or natural aquatic environments. Effective methods for monitoring these ARB in the environment are needed to understand their influence on dissemination of ARB and ARGs, but standard methods have not been developed. This systematic review considers peer-reviewed papers where the ARB above were cultured from wastewater or surface water, focusing on the accuracy of current methodologies.

**Recent Findings:**

Recent studies suggest that many clinically important ARGs were originally acquired from environmental microorganisms. *Acinetobacter*, *Aeromonas,* and *Pseudomonas* species are of interest because their ability to persist and grow in the environment provides opportunities to engage in horizontal gene transfer with other environmental bacteria. Pathogenic strains of these organisms resistant to multiple, clinically relevant drug classes have been identified as an urgent threat. However, culture methods for these bacteria were generally developed for clinical samples and are not well-vetted for environmental samples.

**Summary:**

The search criteria yielded 60 peer-reviewed articles over the past 20 years, which reported a wide variety of methods for isolation, confirmation, and antibiotic resistance assays. Based on a systematic comparison of the reported methods, we suggest a path forward for standardizing methodologies for monitoring antibiotic resistant strains of these bacteria in water environments.

**Supplementary Information:**

The online version contains supplementary material available at 10.1007/s40572-023-00393-9.

## Introduction

Antibiotic resistance is a global human health crisis. According to the US Centers for Disease Control (CDC), nearly 3 million infections by antimicrobial resistant bacteria (ARB) and fungi occur in the USA every year, resulting in over 35,000 deaths [1]. Environmental dimensions to the antibiotic resistance problem are increasingly under scrutiny [2, 3], leading to greater recognition of the value that environmental monitoring could provide for protection of public health [3, 4]. In particular, aquatic environments are suspected to serve as both a reservoir and pathway for dissemination of ARB and antibiotic resistance genes (ARGs) observed in clinical settings [5]. Aquatic environments receive a vast array of anthropogenic inputs, which include wastewater, recycled water, and stormwater, positioning them to play a key role in the evolution and dissemination of ARB. Aquatic environments also present several relevant routes of human exposure, including recreational use, occupational exposure, irrigation of food crops and recreational fields, food production (e.g., vegetable cultivation, aquaculture), impacted drinking water, and flooding.

Developing a system for monitoring antibiotic resistance in aquatic environments is challenging, due in part to the need to select from among numerous relevant targets. The inextricable issues of selecting an informative target (i.e., one that is a serious health threat and is prevalent enough to detect in the environment) and developing an accurate method for quantifying it that can be used in laboratories and regions with varying levels of resources and technical skills are daunting. While metagenomic analyses can identify phyla and ARGs, they generally lack the ability to confidently link ARGs with potential pathogens and are hampered by a high detection limit [6]. Standard molecular methods such as quantitative polymerase chain reaction (qPCR) are able to quantify target pathogens and ARGs at a lower detection limit, but are also unable to link ARGs to host organisms [7]. Capturing ARB via culture-based methods is advantageous for many reasons, including, confirmation of viability, virulence testing [8], the ability to profile phenotypic and genotypic multi-drug resistance (MDR) [9], and generation of data that can be directly applied to human health risk assessment. However, many media for isolating opportunistic pathogens were developed for clinical applications and do not perform well on environmental samples.

Recently, marked progress has been made in the standardization of methodologies for monitoring viable antibiotic resistant fecal indicator bacteria in the environment. The CDC’s 2019 report on antibiotic resistance identifies extended-spectrum beta-lactamase (ESBL)-producing *Enterobacteriaceae* as a serious threat [1], and standard methods for characterization of ESBL-producing *Enterobacteriaceae* [10] as well as ESBL-producing *Escherichia coli* [11] were published to support integrated One Health (humans-animals-environment) monitoring. While these methods provide a useful basis for global comparison of ESBL-producing fecal bacteria, most *Enterobacteriaceae* and *E. coli* strains are physiologically limited in their ability to survive and grow in aquatic environments [12] and thus are not likely to capture a full picture of the potential for ARB and ARG to be disseminated and possibly amplified in environmental matrices.

**Fig. 1:**
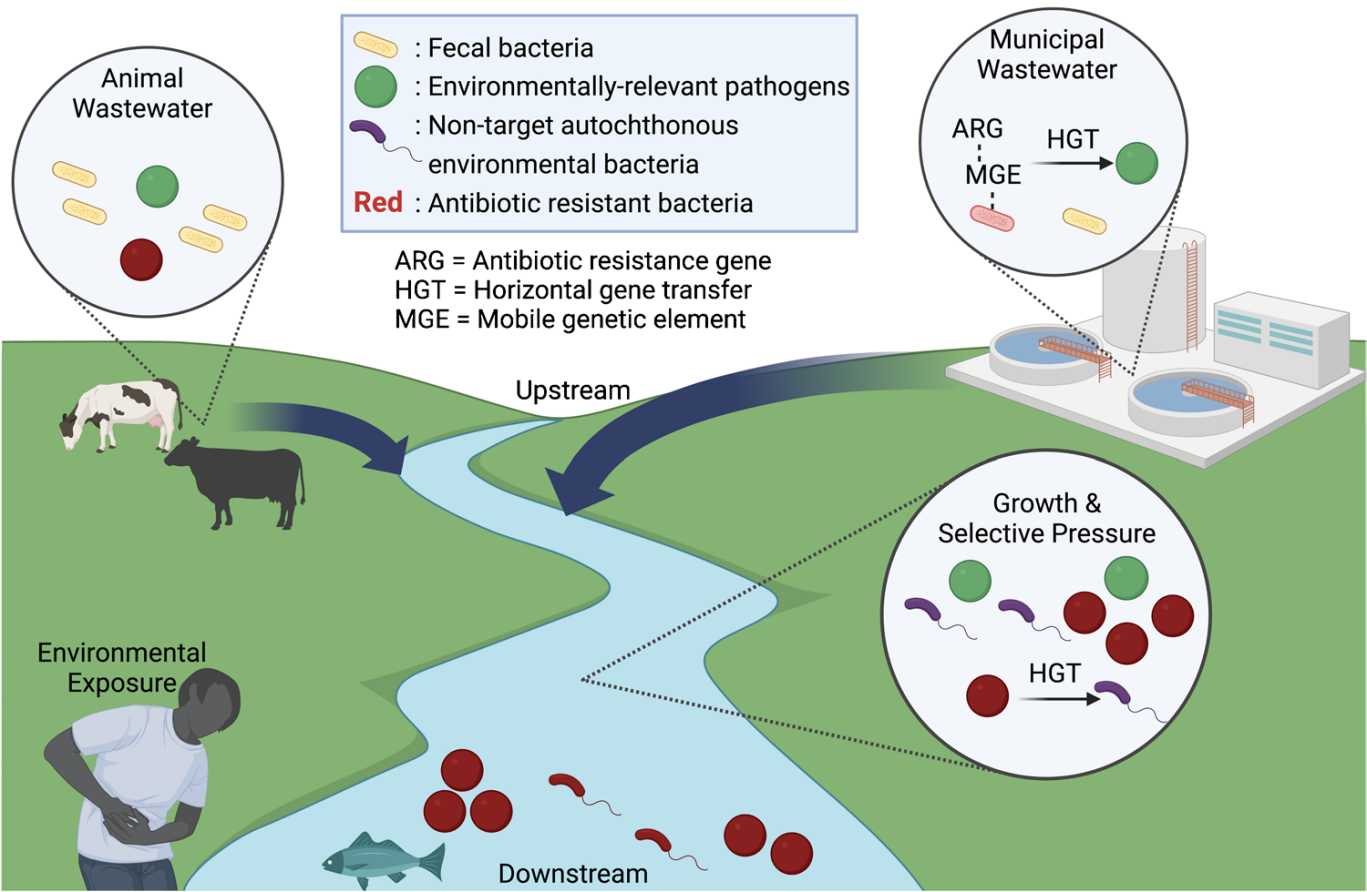
Potential for acquisition of ARGs by environmentally relevant bacteria during wastewater treatment and in impacted surface waters. (Created with BioRender.com by Erin Milligan and Fernando Roman)

An ideal representative of antibiotic resistance potential in aquatic environments would not only be prevalent in environments receiving anthropogenic inputs but would also be capable of persisting and growing in such environments [13]. Extended interaction with the receiving environment would hypothetically afford greater opportunity to engage in horizontal gene transfer of ARGs with a diverse array of other resident bacteria (Fig. 1) [14]. In addition, subpopulations of pathogens harboring ARGs may become dominant in their environment under selection pressure from various pollutants, such as antibiotics, heavy metals, and biocides, which are frequently encountered in livestock and domestic wastewater [15, 16].

Monitoring aquatic bacteria that are also clinically relevant could further strengthen understanding of linkages between environmental reservoirs and pathways of exposure that result in antibiotic-resistant infections in humans. In fact, the origins of several clinically relevant ARGs have been traced back to environmental organisms, including CTX-M and PER, two ESBL-producing ARGs [17, 18•]. There is also evidence that *Acinetobacter baumannii* was the likely origin of dissemination of the metallo-beta-lactamase gene NDM-1 into some *Enterobacteriaceae* [19], and some have hypothesized that most horizontally acquired ARGs originated from environmental microorganisms [20].

Many bacterial genera contain human pathogens that are known to possess environmental niches, including *Acinetobacter, Aeromonas, Arcobacter, Enterococcus, Leptospira, Klebsiella, Pseudomonas, Salmonella, Citrobacter,* and *Vibrio* [21, 22]. Some of these bacteria are of fecal origin, but can also persist in surface water, while others exist as autochthonous populations within aquatic environments. We identified members of *Acinetobacter* spp., *Aeromonas* spp., and *Pseudomonas* spp. as key opportunistic pathogens that have the ability to grow in wastewater and natural aquatic environments and acquire genes that confer multiple antibiotic-resistance (Table 1) and thus have the potential to be versatile targets for culture-based monitoring.

**Table 1:** Diversity of species and human pathogens in environmentally associated genera relevant to this review

Genus	Number of known species	Number of known human pathogens	Most clinically important species	References
*Acinetobacter*	51	20	*Acin. baumannii* *Acin. pitti* *Acin. nosocomialis*	[23]
*Aeromonas*	36	19	*Aero. caviae* *Aero. dhakensis* *Aero. hydrophila* *Aero. veronii*	[24, 25•]
*Pseudomonas*	> 220	9	*P. aeruginosa*	[26, 27]

Certain *Acinetobacter* spp. are an important agent of severe nosocomial infections, including pneumonia and sepsis, but can also cause community-acquired infections [28]. *Acin. baumannii* is most commonly involved in infections, followed by two closely related species (*Acin. pitti* and *Acin. nosocomialis*). *Acin. baumannii* is reported to be more virulent than other species [23, 29]. Severe community-acquired pneumonia caused by of *Acin. baumannii* have been reported in tropical environments, including Asia and Australia [30]. Other community-acquired *Acinetobacter* spp. infections include meningitis, cellulitis, and bacteremia, particularly through wound infections following traumatic injury [31]. Several *Acinetobacter* spp., including *Acin. baumannii* and other pathogenic species, are found in wastewater and impacted freshwater environments [32, 33].

*Aeromonas* spp. cause disease in many hosts, including humans, fish, dogs, cattle, reptiles, and amphibians [34]. Exposure to contaminated fresh or brackish waters is the most common risk factor for human infection [35]. The majority of *Aeromonas* infections are caused by four species: *Aero. caviae, Aero. dhakensis, Aero. veronii,* and *Aero. hydrophila* (Table 1). *Aeromonas* spp. frequently causes severe diarrheal disease; however, they are also associated with mild to severe wound infections, bacteremia, and a variety of extraintestinal infections [25•, 35]. Serious wound infections and sepsis have been reported following leech therapy and emerging fluoroquinolone-resistance has been observed among *Aero. hydrophila* isolated from leeches [36]. *Aeromonas* spp. generally tolerate polluted waters and all but the most extremely concentrated saline waters (> 100%) [34, 37]. Their population levels in aquatic environments tend to peak in warmer months and serious community-acquired infections have also been associated with tropical environments [25•]. *Aeromonas* spp., including those frequently implicated in human disease, are highly abundant in wastewater, rivers, lakes, and reservoirs. Their abundance in surface waters sometimes correlates with fecal indicators, particularly in environments impacted by sewage; however, *Aeromonas* are not considered to be of fecal origin [25•].

Among the diverse array of *Pseudomonas* spp.*, P. aeruginosa* are particularly notorious as agents of multi-antibiotic resistant infections [38]. While widely known as nosocomial pathogens, *P. aeruginosa* also cause community-acquired infections even among healthy individuals. Waterborne *P. aeruginosa* infections can include folliculitis, pneumonia, and otitis externa [39]. *P. aeruginosa* is one of the most common causes of acute external otitis, commonly known as swimmer’s ear [40]. This disease is one of the most common and costly waterborne illnesses in the USA, causing the largest number of emergency room visits [41]. *P. aeruginosa* are commonly found in wastewater and impacted surface waters and can grow in a wide range of aquatic environments. Though considered by some to be ubiquitous in the natural environment, they are only about one-third as likely to be isolated from water environments associated with low human activity compared to those with intense human activity [42••]. *P. aeruginosa* are not typically found in the gastrointestinal tract of healthy individuals but correlate well with fecal indicator bacteria in some studies [43–45].

*Acinetobacter*, *Aeromonas*, and *P. aeruginosa* are well-known for clinically important multi-antibiotic resistant strains. *Acin. baumannii* and *P. aeruginosa* are members of the “ESKAPE” pathogens, a group of life-threatening MDR nosocomial pathogens identified by the Infectious Disease Society of America [46]. MDR *Acin. baumannii* is one of the most difficult to treat Gram-negative infections, and some clinical strains harbor resistance to nearly all conventional antibiotics [47]. The 2019 CDC report, “Antibiotic Resistance Threats in the United States” [1], includes MDR *P. aeruginosa* as a “serious” priority and carbapenemase-producing *Acinetobacter* as “urgent.” The categories “concerning,” “serious,” and “urgent” were first defined by the CDC in the 2013 report [48], with the latter two categories signaling a need for increased monitoring and prevention efforts.

*Aeromonas* spp. and *Pseudomonas* spp. carry many similar intrinsic mechanisms of resistance, including a wide array of beta-lactamases. *P. aeruginosa* can develop carbapenem resistance during therapy, chiefly through a combination of AmpC production and porin change [46]. *P. aeruginosa* and *Aeromonas* spp. may also harbor ESBLs, such as the *Klebsiella pneumoniae* carbapenemase (KPC), which are sometimes associated with the emergence of fluoroquinolone resistance carried on the same plasmid [49, 50]. Members of all three genera also have the capacity to integrate many ARGs on one mobile genetic element (MGE). So-called “resistance islands,” which are assemblages of ARGs acquired through horizontal transfer and integrated into the host chromosome, are also often associated with increased virulence and have been observed in *Acin. baumannii, Aeromonas* spp.*,* and *P. aeruginosa* [24, 51–53]*.*

The objective of this review was to evaluate the current state of culture-based methods employed for enumerating clinically relevant ARB with niches for growth in aquatic environments. We focus on the three genera profiled above: *Acinetobacter*, *Aeromonas*, and *Pseudomonas*. Members of each genus are biofilm-forming opportunistic pathogens known to have highly elastic genomes that are readily modified by horizontal gene transfer [37, 38]. As they persist or grow in impacted environments, they have the opportunity to acquire and exchange ARGs and thus are likely to be appropriate targets for exploring the evolution of antibiotic resistance in aquatic environments. Through systematic review and analysis of relevant studies conducted in built (e.g., wastewater treatment plants) and natural aquatic environments, we propose a path towards standardized monitoring of these bacteria in wastewater and surface water.

## Methods

We conducted a systematic review of peer-reviewed literature reporting culturing of *Acinetobacter, Aeromonas,* or *Pseudomonas* from wastewater or surface water that also assayed antibiotic resistance among the isolated bacteria. The protocol for this systematic review adhered to the guidelines set forth by the PRISMA Statement (Figure S1). The literature search was conducted using an English language search in both Web of Science and PubMed spanning Jan 1, 2000 to May 1, 2020. A tiered search strategy was employed for each target organism that combined topic searches for studies that (1) assessed antibiotic resistance, (2) focused on wastewater, recycled water, or surface water environments, (3) used culture-based methods, and (4) focused on the genera of interest. The search terms used are included in the supplemental information (Table S1).

Studies were excluded if they focused specifically on biosolids, drinking water, or ballast water. Studies that did not use selective media or first used a non-selective enrichment step, did not evaluate antibiotic resistance in isolates, or used isolates of unknown origin or with no culturing details were also excluded. Selective media were defined as media designed for recovery of the target organism, which include components that inhibit the growth of non-target bacteria. Selective media may possess additional component(s) that clearly distinguish the target organism from other bacteria known to grow on the media. Aquaculture and other animal farming studies were excluded from the search, except for cases where surface water was under direct influence of animal wastewater. After removal of duplicates, the original search returned 810 papers; 750 were excluded for the reasons outlined above. Of the 60 remaining papers included in this review, 11 targeted *Acinetobacter*, 26 *Aeromonas*, and 26 *Pseudomonas*. No studies that analyzed recycled water were identified, therefore analysis of this environment was removed from further consideration. Secondary searches were conducted to address other gaps identified in the review. In particular, many of the selective media employed by the included studies have been validated for drinking water, but not wastewater or surface water. Thus, drinking water studies were used as a starting point for comparison of these media. Such articles are discussed in the text but are not included in the figures or summary statistics.

## Results

### Isolation and Confirmation of Genus and Species

#### Experimental Design and Methods for Isolate Confirmation

Given the complexity of environmental samples (e.g., ~ 650 genera typically present in wastewater effluent [54]), the potential for non-target organisms to grow on selective-differential media is substantial. Further, available media were generally developed for clinical samples, which have a more limited and distinct spectrum of bacteria that can interfere with isolation of the target. The decision to confirm to genus versus species depends upon the research question, i.e., one may be concerned with enumerating only known pathogens, or one may wish to capture all members of a given genus (Table 2). Parameters for determining resistance to various antibiotics can be specific to specific classes, species, or strains of bacteria. Furthermore, many bacteria are intrinsically resistant to certain antibiotics, therefore omission of confirmation procedures contributes to the risk of erroneously high estimates of specific ARB. In some cases, however, genus-level confirmation may be sufficient, or even desirable to achieve the research/monitoring objectives. For example, one study assessed the distribution of MGEs among *Aeromonas* isolated from polluted and non-polluted waters [55].

**Table 2:** Examples of the objectives of studies examining antibiotic resistance in *Acinetobacter*, *Aeromonas*, or *Pseudomonas* and methods used for isolate characterization at all levels. *MLSA* multi-locus sequence analysis; *MALDI-TOF MS* matrix-assisted laser desorption/ionization-time of flight mass spectrometry

Target organism	Stated purpose of study	Methods for isolate characterization	References
*Acin. baumannii*	To investigate the connection between *Acin. baumannii* isolates implicated in hospital outbreaks and environmental isolates from hospital and urban wastewater and river	Confirmation to species by VITEK 2 (biochemical tests), MALDI-TOF MS, and *rpoB* sequencing Genetic relatedness among isolates was determined using MLSA targeting *gltA, gyrB, gdhB, recA,cpn60, gpi* and *rpoD*	[56]
*Acin. baumannii*	To determine the propagation and fate of *Acin. baumannii* through the wastewater treatment plant	Confirmation to species by MALDI-TOF MS The genetic relatedness among a subset of isolates was determined using WGS and core genome-MLSA	[57]
*Aeromonas* spp.	To identify similarity of MGEs among strains isolated from polluted and non-polluted waters, and to determine whether these MGEs were transferable	Fifty *Aeromonas* spp. colonies per sample were identified to the species level using MALDI-TOF MS	[55]
*Aeromonas* spp.	To compare *Aeromonas* spp. antibiotic susceptibility and potential for virulence in wastewater effluents and recipient waters over three years	Isolates were identified to the species level by sequencing *gyrB*	[58]
*Pseudomonas* spp.	To measure the incidence of *Pseudomonas* spp. in wastewater and freshwater environments, and to assess the prevalence of ARGs in the isolates	Isolates were confirmed to the genus level by PCR Preliminary identification of species was carried out using API 20NE (biochemical tests), followed by PCR for *P. aeruginosa, P. fluorescens,* and *P. putida*	[59]
*P. aeruginosa*	To determine the risk of dissemination of antibiotic-resistant *P. aeruginosa* from the hospital to the environment via the wastewater network	Up to five colonies per plate were confirmed to the species level using API 32 GN (biochemical tests) Strain relatedness was assessed using pulsed-field gel electrophoresis, and evolutionary relationships among isolates was investigated using MLSA targeting *csA, aroE, guaA, mutL, nuoD, ppsA,* and *trpE*	[60]

Most of the studies reviewed here carried out conventional phenotypic tests for isolate identification (76.6%), including the fully or semi-automated systems API 20 NE, API 32 GN, BD Phoenix ID, MicroScan autoSCAN-4, MicroStation ID, and VITEK 2 ID (Figure S3). Further, 35% of studies relied solely on phenotypic methods for isolate confirmation. Phenotypic tests can be useful for presumptive identification; however, most of them are error-prone [61, 62], therefore, a molecular confirmation step increases confidence in identification.

PCR was used as a confirmation method in 16 (26.7%) studies. Three *Acinetobacter* studies used PCR for confirmation. Two amplified the *Acinetobacter* 16S rRNA gene [63] for confirmation to the genus level, and another targeted OXA-51-like to identify *Acin. baumannii* [64]. Two *Aeromonas* studies targeted the *gyrB* gene, but used different primer sets [65, 66] for confirmation of *Aeromonas* spp., one targeted *aroA* [67] and one targeted the *Aeromonas* spp. specific virulence genes *aerA* and *hylH* [68]. One *Pseudomonas* study targeted the 16S rRNA gene to confirm to the genus level, another targeted *oprI* [69], and another targeted *ecfX* [70], *gyrB* [71], and *toxA* [72]. Three studies confirmed *P. aeruginosa* by targeting variable regions 2 and 8 of the 16S rRNA gene as described by Spilker et al. [73], one study targeted the 23S gene [74], and another targeted *gyrB* [75]. Lastly, one study used the TaqMan® *Pseudomonas aeruginosa* Detection Kit, which employs real-time PCR using proprietary primers and probes.

Twenty-five of twenty-six *Aeromonas* studies targeted all members of the genus, with 73.1% speciating some portion of the recovered isolates. However, 31.6% of these relied on phenotypic tests and 10.5% relied on 16S rRNA gene sequencing for species identification*.* 16S rRNA gene sequencing is generally sufficient for genus level confirmation of the three organisms studied here, but can lead to error in speciation of *Aeromonas*, which tend to have high sequence similarity among species [76]. An additional challenge to all confirmation methods is that classification of potential target bacterial phyla is evolving as understanding of species diversity and environmental distribution grows. A 2015 study reassessed the phylogenetic identity of the 44 *Aeromonas* genomes deposited in the National Center for Biotechnology Information (NCBI) database, finding 12 mislabeled genomes, 11 of which were originally identified as *Aero. hydrophila*. Nine were reclassified as *Aero. dhakensis* [76], which was not described until 2013. While isolation of *Aeromonas* from built and natural aquatic environments has been focused on *Aero. hydrophila,* studies in the last decade have shown *Aero. dhakensis* to be more virulent [77] and *Aero. veronii* to be more prevalent in wastewater effluent and surface water [58]. In such cases sequencing of other housekeeping genes that evolve faster than the 16S rRNA gene and correspond to higher variability among closely-related species may be a better choice. Sequencing of the housekeeping gene *gyrB* was used by six *Aeromonas* studies for speciation, while three *Acinetobacter* studies sequenced *rpoB*.

The limitations of automated identification methods have been noted for *Acinetobacter* spp. as well. The three most clinically important *Acinetobacter* spp. are also closely related to the environmental bacterium *Acin. calcoaceticus*, which is rarely implicated in disease. The four species are often grouped together as the *Acin. calcoaceticus-baumannii* complex due to the difficulty in distinguishing the species from one another [78]. Researchers should be careful to use the higher resolution methods discussed when targeting *Acin. baumannii.* Several studies focused specifically on *Acin. baumannii* or *P. aeruginosa*. A frequent theme among studies targeting a specific pathogen is a focus on source tracking, e.g., comparing strains associated with hospital outbreaks to isolates found in the wastewater network and receiving environments (Table 2). This type of study requires characterization of isolates beyond the species level using a method such as multi-locus sequence analysis (MLSA) to determine genetic relatedness, which involves PCR-based amplification and sequencing of several housekeeping genes.

An alternative molecular identification method for speciation is matrix-assisted laser desorption/ionization-time of flight mass spectrometry (MALDI-TOF MS), which is based on amino acid rather than nucleic acid sequences [79]. User-friendly automated MALDI-TOF MS platforms are available but can have high capital costs and expensive maintenance contracts [80]. MALDI-TOF MS has been shown to accurately identify 96.7% of clinical *Aero. dhakensis* isolates [81]; however, as of 2020 *Aero. dhakensis* and other newly discovered species were not yet included in the MALDI Biotyper commercial database [24, 82]. *Acin. baumannii*, *Acin. pittii*, and *Acin. nosocomialis* were identified by MALDI-TOF MS, as well as two novel pathogens *Acin. seifertii* and *Acin. dijkshoorniae,* at 96.8–99.6% accuracy after adding the novel species to the MALDI Biotyper database [83]. MALDI-TOF MS has been used to identify specific metallo-beta-lactamase-producing strains of *P. aeruginosa* [84].

#### Characteristics and Performance of Selective-Differential Media for Culturing *Acinetobacter*, *Aeromonas*, and *Pseudomonas*

**Fig. 2:**
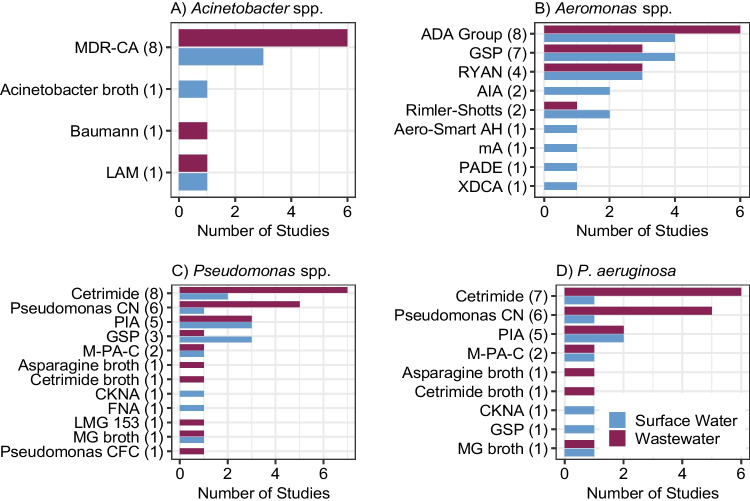
Summary of selective media used for isolation from surface water (blue) and wastewater (red) (total number in parentheses) of A) *Acinetobacter* spp., B) *Aeromonas* spp., C) *Pseudomonas* spp., and D) *Pseudomonas aeruginosa,* where panel C includes all media used in the *Pseudomonas* studies, whereas panel D includes only studies that specifically selected for *P. aeruginosa* on differential media (e.g., via differential colony morphology). Isolation media is solid media (agar) unless denoted as “broth”. Detailed information about each medium is included in SI Table 2

At least three different culture media were used for each of the selected environmental bacterial targets (Fig. 2) and incubation times and temperatures often varied for a given medium. The specificity of the media for their intended bacterial target varied widely (Table 3). *Acinetobacter* spp. (*n* = 11 studies) was cultured using commercial chromogenic media, i.e., CHROMagar Acinetobacter, in 72.7% of studies. This medium is intended for clinical microbiology use, e.g., stool, urine, wounds, perineal and rectal samples. The eight studies using CHROMagar Acinetobacter included the addition of the proprietary “MDR” supplement CR102 (MDR-CA), which selects for carbapenem-resistant strains. One study each used Acinetobacter broth and Baumann agar, which are formulated the same, and one study used Leeds Acinetobacter medium (LAM).

**Table 3:** Summary of papers that reported confirmation frequency of bacterial targets (n = 9 studies). Studies that compared two confirmation methods are listed twice in the table

Organism	Isolation media	Source of isolates	Confirmati-on rate (%)	Confirmation method	Phylogen-etic level	Proportion of isolates tested	Refe-rences
*Acinetobacter* spp.	MDR-CA	River water and sediment	31.3	API 20 NE	Genus	100%	[32]
*Aeromonas* spp.	RYAN	Wastewater effluent	46.0	Sequenced *gyrB*, *radA*	Genus	100%	[85]
*Aeromonas* spp.	RYAN	Estuary	33.0	Sequenced *gyrB*, *radA*	Genus	100%	[85]
*Aeromonas* spp.	RYAN	Surface water and wastewater	74–87.7	MALDI-TOF MS	Genus	5–8 per sample	[86]
*Aeromonas* spp.	ADA	Hospital waste- water effluent	75.0	Fatty acid methyl ester	Genus	5 per plate	[87]
*Aeromonas* spp.	AIA	Urban playa lake	100	Biolog MicroLog	Genus	100%	[88]
*Aeromonas* spp.	AIA	Urban playa lake	100	Sequenced *gyrB*	Genus	2.9%	[88]
*Aeromonas* spp.	ADA-V	Natural reservoir	81.3	Biochemical tests	Genus	10%	[13]
*P. aeruginosa*	Pseudomonas CN	Natural reservoir	100	Biochemical tests	Species	10%	[13]
*P. aeruginosa*	Pseudomonas CN	Natural reservoir	100	PCR of 16S rRNA gene variable regions 2 and 8	Species	10%	[13]
*P. aeruginosa*	Pseudomonas CN	River	93.0	TaqMan® *Pseudomonas aeruginosa* Detection Kit	Species	100%	[89]
*P. aeruginosa*	Cetrimide agar	Hospital waste- water effluent	93.1	Biochemical tests	Species	NA^*^	[90]
*P. aeruginosa*	Cetrimide agar	Hospital waste- water effluent	86.2	Sequenced 16S rRNA gene	Species	NA^*^	[90]
*P. aeruginosa*	PIA	Surface water and wastewater	73.3	PCR of *gyrB*	Species	3–5 per sample	[91]

^*^Proportion of isolates tested was not reported

We included 26 studies in which *Aeromonas* spp. were isolated from wastewater and surface water in this review. Seven studies wherein *Aeromonas* were isolated (26.9%) used ampicillin-dextrin agar with various combinations and concentrations of antibiotics to optimize selectivity (ADA Group in Fig. 2). This method differs from typical methods applied for isolating *Aeromonas* spp. from clinical samples, where inclusion of ampicillin is not recommended because some *Aeromonas* spp. are sensitive to ampicillin [35]. Glutamate starch phenol red agar (GSP) was also used in seven studies. GSP can also detect *Pseudomonas* spp., which are differentiated from *Aeromonas* spp. by the inability of *Pseudomonas* to use starch as a carbon source. Ryan’s Aeromonas medium (RYAN) was used in four (15.4%) studies. Rimler-Shotts medium was used in two studies, one of which assayed both surface water and wastewater, and two surface water studies used Aeromonas Isolation agar (AIA). One study each used Aero-Smart AH, ampicillin-trehalose agar (mA), pril-ampicillin-dextrin-ethanol agar (PADE), and xylose deoxycholate citrate agar (XDCA).

*Pseudomonas* were most frequently cultured on cetrimide agar, which is selective for *P. aeruginosa* (30.8% of 26 studies). The addition of the antiseptic cetrimide to King’s medium A increased specificity of the medium [92], which was further improved by the addition of highly purified cetrimide at 0.03% [93, 94]. Pseudomonas CN, which consists of Pseudomonas agar base with the addition of cetrimide and nalidixic acid, was the second most used isolation media (19.2%). Pseudomonas agar can differentiate between *P. aeruginosa* and other *Pseudomonas* spp. by enhancing pyocyanin pigment production in *P. aeruginosa* (colonies appear blue-green). Pseudomonas Isolation agar (PIA) was the third most frequently used medium for *Pseudomonas* spp. (15.4%). GSP, which cannot differentiate *P. aeruginosa* from other species, was used in three studies (11.5%).

Isolation of *P. aeruginosa* from water types with low background flora, such as treated drinking water, has been standardized by the International Organization for Standardization (ISO) Method 16266 which employs Pseudomonas CN [95]. Only one of the studies that used Pseudomonas CN reported following ISO Method 16266. Isolation from natural and finished surface waters has been standardized by the American Public Health Association’s (APHA) Method 9213 E–F [96], which recommends the use of M-PA-C medium for membrane filtration or asparagine broth for the multiple-tube technique. The M-PA agar base is formulated quite differently (SI Table S2) than King’s Medium and does not include cetrimide; however, the medium does employ kanamycin and nalidixic acid to improve selectivity. One paper included in this review used M-PA-C for both wastewater and surface water and one paper used asparagine broth for wastewater. Additionally, one study each used cephalosporin-fusidin-cetrimide agar (Pseudomonas CFC), Pseudomonas CN with kanamycin (CKNA), Fluorescein Denitrification agar (FNA), *Pseudomonas denitrificans* medium (LMG 153), cetrimide broth, and malachite green (MG) broth.

#### Confirmation of Media Specificity for Target Bacteria

All but one of the studies surveyed here reported a procedure to confirm isolate identification to at least the genus level, but only nine papers (15%; Table 3) reported these data. One *Acinetobacter* study reported a confirmation rate of 31.3% for sediment and water isolates recovered on MDR-CA. One study reported a 100% confirmation rate of isolates from AIA by biochemical tests and sequencing of the *gyrB* gene; however, only three isolates (2.9%) were sequenced (Table 3). Reported confirmation rates for RYAN agar in wastewater and surface water ranged from 33 to 87.7% (Table 3). One study reported a confirmation rate of 75.0% for ADA, while another reported 81.3% for ADA-V (Table 3). The US Environmental Protection Agency Method 1605 [97] recommends use of ADA with the addition of vancomycin (ADA-V) for isolation of *Aeromonas* spp*.* from drinking water. One study showed that the addition of irgasan (ADA-VI) reduced non-*Aeromonas* growth and did not affect recovery of presumptive *Aeromonas* spp. (determined by colony morphology only) from surface water samples [98].

Pseudomonas CN and cetrimide agar were generally specific for *P. aeruginosa.* One study reported a confirmation rate of 100% for *P. aeruginosa* isolated on Pseudomonas CN via biochemical tests and PCR targeting the 16S rRNA gene (Table 3). Another study reported 93% confirmation on Pseudomonas CN using a TaqMan® *Pseudomonas aeruginosa* Detection Kit, which employs real-time PCR using proprietary primers and probes (Table 3). One study confirmed isolates from cetrimide agar by biochemical tests (93.1% confirmed) and sequencing of the 16S rRNA gene (82.6% confirmed) (Table 3). In 1972, Lilly and Lowbury [99] compared cetrimide agar to Pseudomonas CN and found that the addition of nalidixic acid to cetrimide agar greatly improved selectivity and yield for *P. aeruginosa.* They observed that some Gram-negative non-target bacteria, especially *Klebsiella* spp. and *Providencia* spp., can grow on cetrimide agar without nalidixic acid [99]. The addition of kanamycin to Pseudomonas CN (CKNA) has been investigated on clinical samples and showed improved sensitivity (88.2%) and specificity (99.2%) over Pseudomonas CN (81.3% and 98.4%, respectively), with confirmation performed using the Vitek System [100]. Lastly, one study using PIA as the isolation medium reported a confirmation rate of 73.3% using PCR of *gyrB* (Table 3).

### Antibiotic Susceptibility Testing

All studies meeting inclusion criteria for this review performed some type of antibiotic resistance characterization by phenotype or genotype. Phenotypic antibiotic susceptibility testing may be performed during the initial selection process by isolating the target bacteria in the presence of an antibiotic of interest, or it may be carried out post-isolation on individual isolates with one or more antibiotics. In either case, it is crucial that the antibiotic(s) and concentration(s) used are appropriate for the target bacteria. When antibiotics are used in the primary isolation step it is useful to include a no-antibiotic treatment so that the proportion of antibiotic-resistant bacteria can be determined. A minority of the studies reviewed here used an antibiotic in the primary isolation step (15 of 60, or 25%). Eight studies used the CHROMagar MDR supplement for *Acinetobacter* spp. isolation, while another *Acinetobacter* study used ampicillin, gentamicin, and tetracycline independently and altogether. Three *Aeromonas* studies used ciprofloxacin, tetracycline, and oxytetracycline for isolation. One study each used imipenem and ciprofloxacin in the isolation of *P. aeruginosa*.

The majority of included studies (65%) used Kirby-Bauer disk diffusion for post-isolation antibiotic susceptibility testing. Commercial systems for determining minimum inhibitory concentrations (MIC) were most frequently reported for *Acinetobacter* spp., including ETEST and VITEK 2 (each 45.5% of 11 *Acinetobacter* studies). Standards for interpretation of the zone of inhibition (Kirby-Bauer) and MIC have been promulgated by the European Committee on Antimicrobial Susceptibility Testing (EUCAST) and the Clinical and Laboratory Standards Institute (CLSI). A majority of *Aeromonas* spp. and *Pseudomonas* spp. studies used CLSI standards (54.2% of 24 studies for each) while EUCAST solely or supplemented by CLSI was most frequently reported for *Acinetobacter* spp. testing (54.5% of 11 studies). Six studies (10.7% of 56 studies) did not cite how MICs were interpreted.

Another approach to infer antibiotic resistance of cultured isolates is by PCR analysis of specific genes or whole genome sequencing (WGS) to identify ARGs and MGEs. A caveat of these approaches is that the presence of a given ARG does not confirm that the phenotype will be expressed [101]. Included studies largely targeted beta-lactamase ARGs and integron-integrase MGEs (Table 4). A number of *Acinetobacter* studies targeted only OXA genes. There is growing evidence that *Acin. baumannii* can carry intrinsic resistance to carbapenem antibiotics through OXA-51-like genes [102]. Turton et al. [103] found that these genes only conferred clinically appreciable carbapenem resistance when the insertion sequence (IS)*Aab*1 lay upstream. Two included studies looked for IS*Aab*1 in their *Acinetobacter* spp. isolates. WGS can be of further use for strain-level identification and to assess the co-occurrence of MGEs, ARGs, virulence factors, and other forms of resistance in a given bacterial strain; however, WGS can be costly and specialized expertise is also necessary to analyze WGS data.

**Table 4:** ARG and MGE targets studied in *Acinetobacter*, *Aeromonas*, and *Pseudomonas* isolates

Organism	Isolation media	Isolation antibiotic	ARGs [MGEs]	References
*Acin. baumannii*	CHROMagar Acinetobacter	MDR supplement	AmpC^a^, IMP^b^, NDM^b^, OXA^c^, [intI1^d^]	[104]
*Acin. baumannii*	CHROMagar Acinetobacter	MDR supplement	OXA^c^	[105]
*Acin. baumannii*	CHROMagar Acinetobacter	MDR supplement	OXA^c^	[106]
*Acin. baumannii*	CHROMagar Acinetobacter	MDR supplement	OXA^c^	[57]
*Acin. baumannii*	CHROMagar Acinetobacter	MDR supplement	OXA^c^	[107]
*Acinetobacter* spp.	CHROMagar Acinetobacter	MDR supplement	IMP^b^, NDM^b^, VIM^b^, GIM^b^, SIM^b^, SPM^b^, OXA^c^, CTX-M^e^, GES^e^, PER^e^, SHV^e^, TEM^e^, VEB^e^, KPC^f^, sul1^g^, sul2^g^, sul2^g^, [intI1^d^, intI2^d^, intI3^d^]	[32]
*Aeromonas* spp.	ADA	None	[intI1^d^]	[55]
*Aeromonas* spp.	GSP	None	TEM^e^, Tet(C)^h^, CARB-2^f^ [intI1^d^, intI2^d^]	[108]
*Aeromonas* spp.	GSP	None	[intI1^d^, intI2^d^, intI3^d^]	[109]
*Aeromonas* spp.	RYAN	None	ACC^a^, CTX-M^e^, FOX^a^, GES^e^, KPC^f^, MOX^a^, OXA^c^, PER^e^, SHV^e^, TEM^e^, VEB^e^, cphA, imiH	[110]
*Aeromonas* spp.	ADA-VI	Tetracycline	Tet(A)^h^, Tet(B)^h^, Tet(C) ^h^, Tet(D)^h^, Tet(E)^h^, Tet(M)^i^, Tet(O)^i^	[58]
*Aero. media*	ADA-VI	None	CTX-M^e^, TEM^e^	[58]
*Pseudomonas* spp.	CFC	None	[intI1^d^, intI2^d^]	[111]
*Pseudomonas* spp.	GSP	None	CTX-M^e^, SHV^e^, TEM^e^	[112]
*P. aeruginosa*	Cetrimide Agar	None	CTX-M^e^, SHV^e^, TEM^e^	[112]
*P. aeruginosa*	Pseudomonas CN	None	aadA^j^, CTX-M^e^, GES^e^, IMP^b^, GIM^b^, NDM^b^, OXA^c^, PER^e^, SIM^b^, SPM^b^, TEM^e^, VEB^e^, VIM^b^ [intI1^d^, intI2^d^]	[113]
*P. aeruginosa*	Cetrimide Agar	None	AmpC^a^	[114]
*P. aeruginosa*	Cetrimide Agar	None	[intI1^d^, intI2^d^, intI3^d^]	[115]

^a^Class C beta-lactamases, ^b^ Class B beta-lactamases, ^c^ Class D beta-lactamases, ^d^ Integron-integrase, ^e^ ESBL, ^f^ Class A beta-lactamases, ^g^ Sulfonamide resistance, ^h^ Tetracycline efflux pumps, ^i^ Tetracycline ribosome protection, ^j^ Aminoglycoside nucleotidyltransferase

### Antibiotic Resistance Trends

**Fig. 3:**
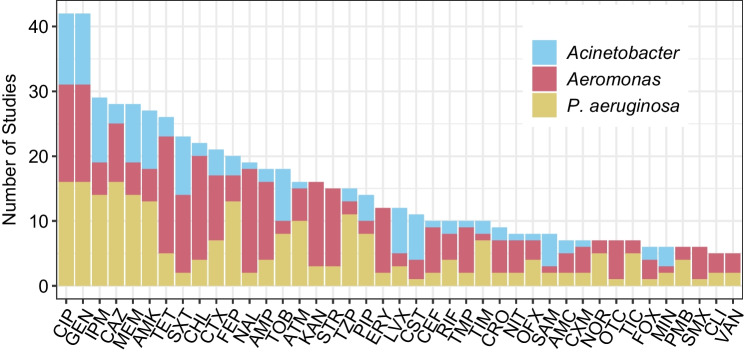
Distribution of the upper 50.^th^ percentile of antibiotics (included in five or more studies) to which phenotypic resistance was assayed for *Acinetobacter* spp. (from *n* = 11 studies), *Aeromonas* spp. (from *n* = 24 studies), and *P. aeruginosa* (from *n* = 19 studies) isolates recovered from wastewater and surface water. Antibiotics are denoted as standard three-letter abbreviations: Ciprofloxacin (CIP), Gentamicin (GEN), Imipenem (IPM), Ceftazidime (CAZ), Meropenem (MEM), Amikacin (AMK), Tetracycline (TET), Sulfamethoxazole/Trimethoprim (SXT), Chloramphenicol (CHL), Cefotaxime (CTX), Cefepime (FEP), Nalidixic acid (NAL), Ampicillin (AMP), Tobramycin (TOB), Aztreonam (ATM), Kanamycin (KAN), Streptomycin (STR), Piperacillin/Tazobactam (TZP), Piperacillin (PIP), Erythromycin (ERY), Levofloxacin (LVX), Colistin (CST), Cephalothin (CEF), Rifampicin (RIF), Trimethoprim (TMP), Ticarcillin/Clavulanic acid (TIM), Ceftriaxone (CRO), Nitrofurantoin (NIT), Ofloxacin (OFX), Ampicillin/Sulbactam (SAM), Amoxicillin/Clavulanic acid (AMC), Cefuroxime (CXM), Norfloxacin (NOR), Oxytetracycline (OTC), Ticarcillin (TIC), Cefoxitin (FOX), Minocycline (MIN), Polymyxin B (PMB), Sulfamethoxazole (SMX), Clindamycin (CLI), Vancomycin (VAN)

Figure 3 summarizes the upper 50^th^ percentile of antibiotics used for post-isolation susceptibility testing among the 82 total antibiotics or antibiotic combinations used across all studies included in this review. Figure S2 shows the frequency of use for all antibiotics used. Ciprofloxacin and gentamicin were among the most frequently used antibiotics for *Acinetobacter* spp., *Aeromonas* spp., and* P. aeruginosa*. Ciprofloxacin, a second-generation fluoroquinolone, is active against Gram-negative and Gram-positive bacteria, and was the 8^th^ most prescribed antibiotic in the USA in 2020 [116]. Gentamicin has been in clinical use since 1963 and also has broad-spectrum activity [117].

Rising resistance to beta-lactam antibiotics among *Acinetobacter* spp. has elevated the importance of carbapenems for intervention against *Acinetobacter* spp. infections, and alternative therapeutic agents for carbapenem-resistant infections are limited. Potential alternatives include fluoroquinolones, aminoglycosides, polymyxins, tigecycline, minocycline, and ampicillin-sulbactam [118]. Generally, the first two classes are not preferred for empiric therapy due to high rates of resistance and are more appropriately used when susceptibility has been established. Additionally, the frequency of resistance to sulbactam in *Acinetobacter* spp. has been increasing [31]. Imipenem and meropenem, among other carbapenems, are on the World Health Organization’s 2019 “Watch” list, which prioritizes stewardship and monitoring programs for these antibiotics [119]. Isolate susceptibility to imipenem does not ensure susceptibility to meropenem and vice versa, thus it is important to test for susceptibility to both antibiotics [118].

*Aeromonas* spp. are typically susceptible to fluoroquinolones, aminoglycosides, carbapenems, and monobactams [24]. Cephalosporins also play an important role in clinical treatment; however, the activity of first-, second-, and third-generation cephalosporins is variable among species [120, 121]. With the exception of *Aero. enteropelogenes*, aeromonads are intrinsically resistant to ampicillin [120, 122], yet 12 studies reported screening for ampicillin resistance in *Aeromonas* isolates. Another example of an inappropriate choice of antibiotics is the use of vancomycin, which is active only against Gram-positive organisms, for both *Aeromonas* and *P. aeruginosa*.

In 2012, Magiorakos et al. [123] published guidelines for determining MDR in *P. aeruginosa* that included a set of antibiotics recommended for testing: ceftazidime, cephalothin, ticarcillin/clavulanic acid, piperacillin/tazobactam, aztreonam, imipenem, meropenem, doripenem, gentamicin, tobramycin, amikacin, netilmicin, ciprofloxacin, levofloxacin, fosfomycin, colistin, and polymyxin B. Many of these are noted to have been captured by the upper 50^th^ percentile of antibiotics tested for *P. aeruginosa* (Fig. 3). While such efforts are useful to standardize monitoring of MDR *P. aeruginosa*, it may also be informative to include novel antibiotics in monitoring schemes. Novel and combined agents that are reserved for MDR *P. aeruginosa* infections, such as ceftolozane-tazobactam, ceftazidime-avibactam, cefiderocol, and imipenem-cilastin-relebactam, were not tested in any of the included studies.

**Fig. 4:**
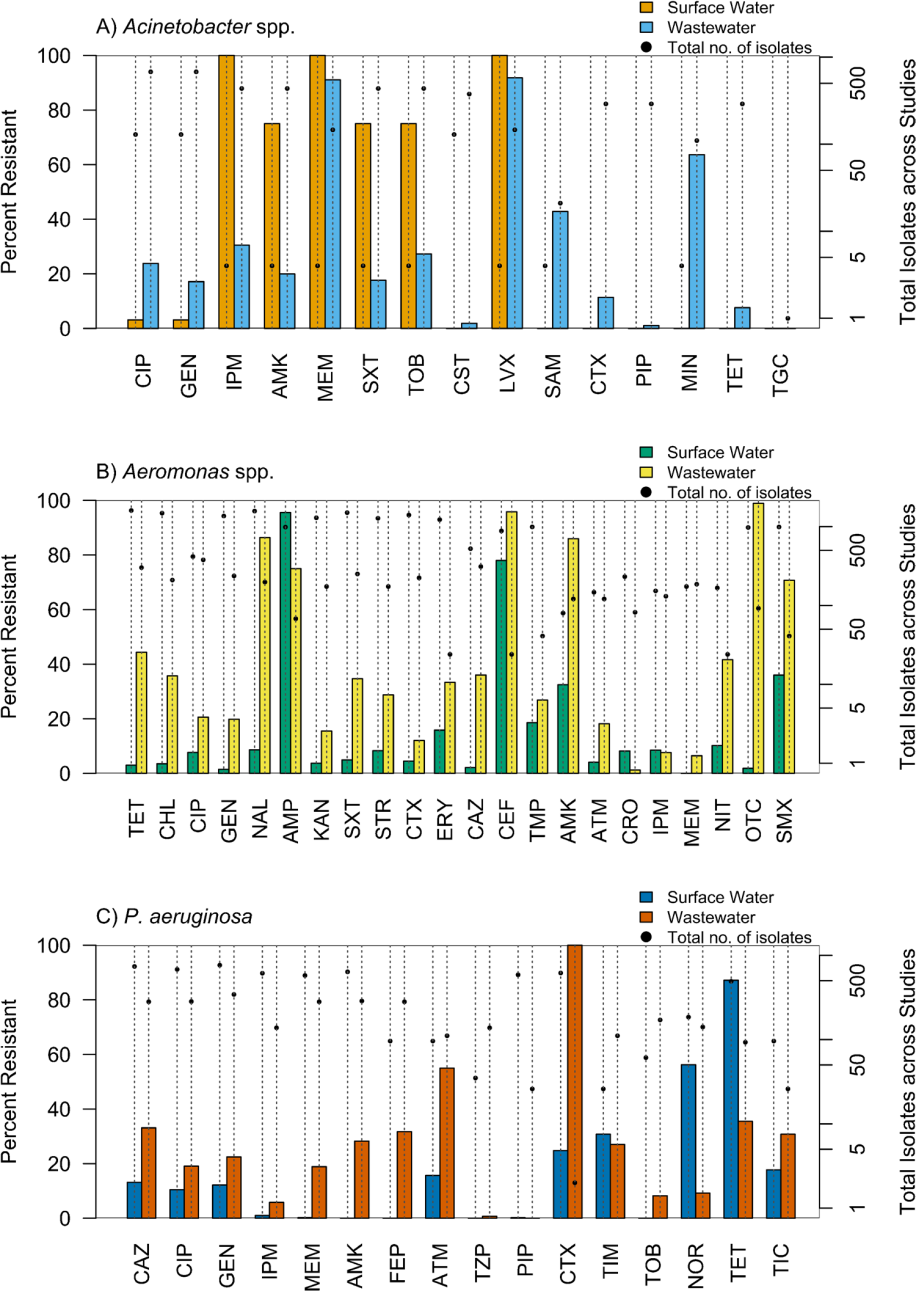
Number of resistant and susceptible isolates enumerated across studies (right axis) and percent resistant (left axis) to the upper 50^th^ percentile of antibiotics tested across studies for A) *Acinetobacter* spp., and upper 25^th^ percentile of antibiotics for B) *Aeromonas* spp. and C) *P. aeruginosa* isolated from wastewater and surface water. In cases where numeric data were not reported (i.e. [57, 85, 108, 124, 125]), corresponding values were estimated from the figures. Isolates that were cultured from environmental samples on media using an isolation antibiotic were excluded apart from *Acinetobacter* spp. isolates captured on CHROMagar Acinetobacter using the MDR supplement. In four cases, susceptibility testing to certain antibiotics was not carried out or reported for any *Acinetobacter* spp. isolates from surface water (cefotaxime, piperacillin, tetracycline, and tigecycline), and thus corresponding data are not plotted

The frequency of resistance to the most frequently used antibiotics for each target organism is compared in Fig. 4*.* Generally, wastewater isolates recovered across studies were more frequently resistant to the antibiotics tested than isolates from surface water. However, *P. aeruginosa* isolated from surface water demonstrated higher resistance rates for some antibiotics, i.e., ticarcillin/clavulanic acid, norfloxacin, and tetracycline. *Acinetobacter* isolates recovered from surface water in one study were resistant to several antibiotics, i.e., imipenem, amikacin, meropenem, sulfamethoxazole/trimethoprim, tobramycin, and levofloxacin. However, only four isolates were recovered and three of those were determined to be the same strain by MLSA. For antibiotics that were tested against a greater number of surface water *Acinetobacter* isolates, resistance was always lower than that of wastewater-derived isolates.

More than 90% of *Acinetobacter* spp. wastewater isolates exhibited resistance to meropenem, while just under 30% were resistant to imipenem. This is unsurprising given that 72.7% of *Acinetobacter* studies used the CHROMagar MDR supplement, which is designed to select for carbapenem resistant isolates. Resistance to the beta-lactams cefotaxime and piperacillin was low. Wastewater isolates exhibited varied resistance to many of the alternative therapeutic agents for MDR *Acinetobacter* infections mentioned above, with notably higher resistance to levofloxacin, ampicillin/sulbactam, and minocycline. Notably, very little resistance was observed for colistin, which is one of the main agents used as a last resort for extensively drug resistant *Acinetobacter* infections [118].

As noted above, *Aeromonas* antibiotic susceptibility varies by species. Only seven included studies reported resistance for speciated *Aeromonas* isolates, some using the methods noted above to have questionable accuracy. Thus, resistance to the top 25% of tested antibiotics is grouped for all species of *Aeromonas* isolated across studies (Fig. 4). *Aero. dhakensis*, *Aero. hydrophila*, and *Aero. caviae* are typically resistant to cephalothin, which may explain the very high resistance to cephalothin in both surface water and wastewater isolates [126]. Isolates showed relatively high susceptibility to ciprofloxacin and third-generation cephalosporins: cefotaxime, ceftazidime, and ceftriaxone, all of which are considered first line empiric therapy for *Aeromonas* infections. In particular, third-generation cephalosporins and/or aminoglycosides are recommended for *Aeromonas* infections from regions with high endemic resistance, such as Bangladesh, where *Aeromonas* spp. are the enteric pathogens with the highest reported rate (82%) of MDR [25•]. Isolates across studies were generally sensitive to aminoglycosides gentamicin, streptomycin, and kanamycin, but resistance to amikacin was high among wastewater isolates (86%). *Aeromonas* spp. are also typically susceptible to tetracycline, but 44% of wastewater isolates were resistant.

Less than 20% of *P. aeruginosa* isolates across studies exhibited resistance to the first and second-line antipseudomonal agents ciprofloxacin, piperacillin/tazobactam, and piperacillin. On the other hand, *P. aeruginosa* isolates from wastewater were more resistant to ceftazidime, cefepime, aztreonam and ticarcillin/clavulanic acid (Fig. 4). Until 2019, the EUCAST breakpoint for aztreonam considered *P. aeruginosa* to be intrinsically resistant [38]. Around 40% of the 76 total *P. aeruginosa* isolates categorized as resistant to aztreonam were tested against a non-inhibitory level of the antibiotic.

## Discussion

Among the sixty studies that met the inclusion criteria for this review, a wide range of methodologies of varying levels of sophistication were reported. The availability of a variety of methods allows researchers the freedom to choose from what is available or cost effective, the degree of automation, and the best method depending on their research question or objectives. On the other hand, the accuracy of the reported methods for identification of the desired genus or species was also widely variable, hampering comparisons and synthesis across studies. Global antibiotic resistance monitoring of water environments requires method standardization in order to achieve consistently accurate measurements of antibiotic-resistant targets that can be compared across studies. We hope that this review takes a substantial step towards informing a common set of methods for culture-based monitoring of three sentinel groups of bacteria that hold particular promise for this purpose because of their clinical relevance and ability to persist, grow, and interact with autochthonous environmental bacteria.

### Towards Standardization

This review demonstrates the lack of standardized methods for the isolation of *Acinetobacter* spp., *Aeromonas* spp., and *Pseudomonas* spp. from wastewater. Moreover, no standardized method for *Acinetobacter* spp. isolation from any water environment was found to exist. Choice of a selective isolation medium hinges on the desired target. As discussed, some researchers may choose to assess all members of a given genus, while others may be interested in targeting a particular species (Table 2). It is important to note that not all species within the three genera focused on in this review are pathogens, and thus the implications for human health may vary or otherwise be unknown for a given target. For example, the genus *Pseudomonas* contains over 220 species, of which only nine are known to be human pathogens (Table 1). *P. aeruginosa*, which is by far the most common cause of *Pseudomonas* infections and a serious MDR threat, was the primary target in two-thirds of *Pseudomonas* studies.

Several culture media were reported which are selective for *P. aeruginosa* (Fig. 2). On the other hand, a majority of *Acinetobacter* (100%) and *Aeromonas* (76.9%) studies used media selective only to the genus level. However, MDR-CA (used in 72.7% of *Acinetobacter* studies) may select for *Acin. baumannii* over other species of *Acinetobacter* due to their intrinsic resistance to carbapenems. A 2020 study by Benoit et al. [127] compared CHROMagar Acinetobacter to LAM (used by just one study in this review) and found that the latter outperformed CHROMagar Acinetobacter for *Acinetobacter* spp. recovery from all tested water matrices other than wastewater effluent. The authors hypothesized that the presence of residual chlorine in the wastewater effluent potentially acted synergistically with antimicrobial reagents in the LAM to inhibit growth. Nine of the eleven *Acinetobacter* studies that met our review criteria were published between 2014 and 2019, indicating an emerging interest in this organism and emphasizing the need for further evaluation of the available selective media to isolate *Acinetobacter* spp. from various environmental matrices.

Following validation of isolation media, confirmation of a representative subset of isolates should be a standard operating procedure. PCR can be applied to confirm a target genus or species, and costs and expertise for conventional PCR are within the reach of many of the larger utilities in developed countries. ISO Method 8199, “Water quality: General guidance on enumeration of micro-organisms by culture” [128], recommends that final reported CFUs be corrected based on the confirmation rates. Without this step, researchers may overestimate the abundance of target organisms in their samples. Research that may require such abundance measures include quantitative microbial risk assessment and evaluation of removal efficiencies via wastewater treatment. Further characterization of isolates may be necessary depending on the research question. As discussed, intrinsic resistance can vary greatly among species within a given genus. *Aero. dhakensis* harbors the AQU-1 gene, which has been shown to confer cefotaxime resistance in derepressed mutants, while other pathogenic species did not exhibit inducible resistance [129••]. There was no reported recovery of *Aero. dhakensis* in any of the studies, which may be why resistance to cefotaxime was infrequently observed among *Aeromonas* spp. (Fig. 4). However, it is not possible to discern how many environmental *Aero. dhakensis* isolates were potentially misidentified in the included studies and simply did not express cefotaxime resistance. Carnelli et al. [55] detected cefotaxime resistant *Aeromonas* isolates in wastewater, which were characterized as *Aero. hydrophila, Aero. media,* and *Aero. caviae* using MALDI-TOF MS. However, their 2012 database only contained 11 *Aeromonas* spp. [130], which makes it very possible one or more of these isolates actually belonged to *Aero. dhakensis*. Thus, caution should be taken to use updated and accurate databases in addition to high resolution methods for speciation of isolates.

Data sharing and open communication will be necessary to advance standardization of methods for the targets identified in this review. Only 15% of studies reported genus or species confirmation rates. Determining confirmation rates is not only essential to provide confidence in estimates of the abundance of specific ARB, but also necessary if findings are to be compared across studies. Additionally, certain reporting standards should be agreed upon. Examples of reporting standards may include the date and location of sampling, water matrix, temperature, pH, and dissolved oxygen of the samples. Supplemental information should include the results from antibiotic susceptibility testing for each isolate, including antibiotic concentration and the diameter of the zone of inhibition, where applicable, as well as the guidelines used to determine susceptibility (e.g., EUCAST vs. CLSI). To encourage and facilitate data sharing and standardization, we developed the Water Antibiotic Resistance Database (WARD), a web-based data repository and analytical tool [131]. For example, researchers can utilize WARD to access and share antibiotic resistance data, metadata, and sampling protocols.

Given their clinical relevance, tendency to carry mobile and clinically relevant forms of MDR, and capability of growth in aquatic environments, *Acinetobacter* spp., *Aeromonas* spp., and *Pseudomonas* spp. are promising targets for monitoring antibiotic resistance in wastewater and surface water. However, it was clear from this review that these organisms have been critically understudied for this purpose. A major challenge to addressing this knowledge gap is the need for standardization of culture and antibiotic resistance profiling methods, which requires more comprehensive reporting of the specificity of culture media. The following methods are suggested as a starting point for potential standardization for wastewater and surface water testing.

1. For the isolation of *Acinetobacter* spp., more research is needed to compare the performance of available media, giving particular attention to the recovery of *Acin. baumannii* versus other species.
2. For *Aeromonas* spp., a direct comparison of GSP agar and ADA-VI is suggested. Confirmation to the genus level can be achieved by PCR amplification of *gyrB*. Speciation of isolates should be performed using molecular methods depending on the research question.
3. For *Pseudomonas* spp., we recommend CN agar to target *P. aeruginosa* due to its documented performance in environmental studies. We also recognize the need to compare the sensitivity and specificity of CN agar to CKNA agar by further evaluating media performance on wastewater and surface water samples. *P. aeruginosa* isolates can be confirmed by PCR amplification of the 16S rRNA gene as described by Spilker et al. [73].

## Conclusions

The spread of antibiotic resistance is a problem that demands global monitoring efforts to extend the usefulness of antibiotic therapy. Built and natural aquatic environments are thought to be key reservoirs and pathways for dissemination of ARB and ARGs into humans and the clinic, and thus are critical environments for antibiotic resistance monitoring. However, global monitoring efforts necessitate the development and utilization of standardized methods in order to reasonably inform decisions in a One Health framework. This systematic review highlights the need for standardized methods for the culture of environmentally and clinically relevant ARB in the environment and recommends a path forward to study *Acinetobacter*, *Aeromonas*, and *Pseudomonas* in wastewater and surface water.

## Supplementary Information

Below is the link to the electronic supplementary material.

Supplementary file1 (DOCX 235 kb)

Supplementary file2 (XLSX 445 kb)

## Data Availability

Data extracted from the cited studies for analysis are available in the Supplementary Materials.

## References

[1] Centers for Disease Control and Prevention (CDC). Antibiotic Resistance Threats in the United States, 2019. Atlanta, GA; 2019.

[2] Smalla K, Cook K, Djordjevic SP, Klümper U, Gillings M (2018). Environmental dimensions of antibiotic resistance: assessment of basic science gaps. FEMS Microbiol Ecol.

[3] Larsson DGJ, Andremont A, Bengtsson-Palme J, Brandt KK, de Roda Husman AM, Fagerstedt P (2018). Critical knowledge gaps and research needs related to the environmental dimensions of antibiotic resistance. Environ Int.

[4] JPIAMR. Strategic Research and Innovation Agenda on Antimicrobial Resistance, version 2021. 2021.

[5] Hernando-Amado S, Coque TM, Baquero F, Martínez JL (2019). Defining and combating antibiotic resistance from One Health and Global Health perspectives. Nat Microbiol Nature Publishing Group.

[6] Liguori K, Keenum I, Davis BC, Calarco J, Milligan E, Harwood VJ (2022). Antimicrobial Resistance Monitoring of Water Environments: A Framework for Standardized Methods and Quality Control. Environ Sci Technol.

[7] Keenum I, Liguori K, Calarco J, Davis BC, Milligan E, Harwood VJ, et al. A framework for standardized qPCR-targets and protocols for quantifying antibiotic resistance in surface water, recycled water and wastewater. Crit Rev Environ Sci Technol. 2022;4395–419. 10.1080/10643389.2021.2024739.

[8] Lagier JC, Dubourg G, Million M, Cadoret F, Bilen M, Fenollar F (2018). Culturing the human microbiota and culturomics. Nat Rev Microbiol.

[9] Lagier J-C, Dubourg G, Amrane S, Raoult D (2017). Koch Postulate: Why Should we Grow Bacteria?. Arch Med Res.

[10] Marano RBM, Fernandes T, Manaia CM, Nunes O, Morrison D, Berendonk TU, et al. A global multinational survey of cefotaxime-resistant coliforms in urban wastewater treatment plants. Environ Int. 2020;144. 10.1016/j.envint.2020.106035. 10.1016/j.envint.2020.106035 32835921

[11] World Health Organization (WHO). WHO Integrated Global Surveillance on ESBL-producing E. coli Using a “One Health” Approach. World Health Organization; 2021.

[12] Zhi S, Stothard P, Banting G, Scott C, Huntley K, Ryu K, et al. Characterization of water treatment-resistant and multidrug-resistant urinary pathogenic Escherichia coli in treated wastewater. Water Res. 2020;182. 10.1016/j.watres.2020.115827. 10.1016/j.watres.2020.115827 32580076

[13] Blasco MD, Esteve C, Alcaide E (2008). Multiresistant waterborne pathogens isolated from water reservoirs and cooling systems. J Appl Microbiol.

[14] Rizzo L, Manaia C, Merlin C, Schwartz T, Dagot C, Ploy MC (2013). Urban wastewater treatment plants as hotspots for antibiotic resistant bacteria and genes spread into the environment: A review. Sci Total Environ.

[15] Tello A, Austin B, Telfer TC (2012). Selective pressure of antibiotic pollution. Environ Health Perspect.

[16] Baquero F, Tedim AP, Coque TM. Antibiotic resistance shaping multi-level population biology of bacteria. Front Microbiol. 2013;4. 10.3389/fmicb.2013.00015. 10.3389/fmicb.2013.00015 PMC3589745 23508522

[17] Humeniuk C, Arlet G, Gautier V, Grimont P, Labia R, Philippon A (2002). β-lactamases of Kluyvera ascorbata, probable progenitors of some plasmid-encoded CTX-M types. Antimicrob Agents Chemother.

[18.•] Ebmeyer S, Kristiansson E, Larsson DGJ (2019). PER extended-spectrum β-lactamases originate from Pararheinheimera spp. Int J Antimicrob Agents.

[19] Bonnin RA, Poirel L, Nordmann P (2014). New Delhi metallo-β-lactamase-producing Acinetobacter baumannii: A novel paradigm for spreading antibiotic resistance genes. Future Microbiol.

[20] Martínez JL. Ecology and Evolution of Chromosomal Gene Transfer between Environmental Microorganisms and Pathogens. Microbiology Spectrum. 2017;6. 10.1128/microbiolspec.mtbp-0006-2016. 10.1128/microbiolspec.mtbp-0006-2016 PMC11633556 29350130

[21] Pruden A, Ashbolt N, Miller J. Overview of issues for water bacterial pathogens. In: Rose J, Jiménez-Cisneros B, Pruden A, Ashbolt N, Miller J, editors. Water and Sanitation for the 21st Century: Health and Microbiological Aspects of Excreta and Wastewater Management (Global Water Pathogen Project). E. Lansing, MI: Michigan State University, UNESCO; 2019. 10.14321/waterpathogens.20.

[22] Finley RL, Collignon P, Larsson DGJ, Mcewen SA, Li XZ, Gaze WH (2013). The scourge of antibiotic resistance: The important role of the environment. Clin Infect Dis.

[23] Shin B, Park W (2017). Antibiotic resistance of pathogenic Acinetobacter species and emerging combination therapy. J Microbiol.

[24] Fernández-Bravo A, Figueras MJ (2020). An update on the genus Aeromonas: Taxonomy, epidemiology, and pathogenicity. Microorganisms.

[25.•] Figueras Salvat MJ, Ashbolt N. Aeromonas. In: Rose JB, Jiménez-Cisneros B, Pruden A, Ashbolt N, Miller J, editors. Water and Sanitation for the 21st Century: Health and Microbiological Aspects of Excreta and Wastewater Management (Global Water Pathogen Project). E. Lansing, MI: Michigan State University, UNESCO; 2019. 10.14321/waterpathogens.21. **Comprehensive review of *****Aeromonas***** spp. environmental occurrence, transmission, and disease association.**

[26] Iglewski BH. Pseudomonas. In: Baron S, editor. Medical Microbiology. 4th ed. Galveston, TX: University of Texas Medical Branch at Galveston; 1996. 21413252

[27] Lalucat J, Mulet M, Gomila M, García-Valdés E. Genomics in bacterial taxonomy: Impact on the genus pseudomonas. Genes (Basel). 2020;11. 10.3390/genes11020139. 10.3390/genes11020139 PMC7074058 32013079

[28] Joly-Guillou ML (2005). Clinical impact and pathogenicity of Acinetobacter. Clin Microbiol Infect.

[29] Chusri S, Chongsuvivatwong V, Rivera JI, Silpapojakul K, Singkhamanan K, McNeil E (2014). Clinical outcomes of hospital-acquired infection with Acinetobacter nosocomialis and Acinetobacter pittii. Antimicrob Agents Chemother.

[30] Anstey NM, Currie BJ, Withnall KM (1992). Community-Acquired Acinetobacter Pneumonia in the Northern Territory of Australia. Clin Infect Dis.

[31] Wong D, Nielsen TB, Bonomo RA, Pantapalangkoor P, Luna B, Spellberg B (2017). Clinical and pathophysiological overview of Acinetobacter infections: A century of challenges. Clin Microbiol Rev Am Soc Microbiol.

[32] Maravić A, Skočibušić M, Fredotović Ž, Šamanić I, Cvjetan S, Knezović M (2016). Urban riverine environment is a source of multidrug-resistant and ESBL-producing clinically important Acinetobacter spp. Environ Sci Pollut Res.

[33] Adewoyin MA, Okoh AI (2018). The natural environment as a reservoir of pathogenic and non-pathogenic Acinetobacter species. Rev Environ Health.

[34] Janda JM, Abbott SL (2010). The genus Aeromonas: Taxonomy, pathogenicity, and infection. Clin Microbiol Rev.

[35] Morris JG, Horneman A. Aeromonas infections. In: Calderwood SB, Baron EL, editors. UpToDate. Waltham, MA: UpToDate Inc.; 2022. Available from: https://www.uptodate.com. Accessed 14 Apr 2022.

[36] Patel KM, Svestka M, Sinkin J, Ruff P (2013). Ciprofloxacin-resistant Aeromonas hydrophila infection following leech therapy: A case report and review of the literature. J Plast Reconstr Aesthet Surg.

[37] Hazen TC, Fliermans CB, Hirsch RP, Esch GW (1978). Prevalence and Distribution of Aeromonas hydrophila in the United States. Appl Environ Microbiol.

[38] Horcajada JP, Montero M, Oliver A, Sorlí L, Luque S, Gómez-Zorrilla S, et al. Epidemiology and treatment of multidrug-resistant and extensively drug-resistant Pseudomonas aeruginosa infections. Clin Microbiol Rev. American Society for Microbiology; 2019;32. 10.1128/CMR.00031-19. 10.1128/CMR.00031-19 PMC6730496 31462403

[39] Kanj SS, Sexton DJ. Epidemiology, microbiology, and pathogenesis of Pseudomonas aeruginosa infection. In: Calderwood SB, Hall KK, editors. UpToDate. Waltham, MA: UpToDate Inc.; 2022 [cited 2022 Apr 16]. Available from: https://www.uptodate.com. Accessed 17 Apr 2022.

[40] Schaefer P, Baugh RF (2012). Acute Otitis Externa: An Update. Am Fam Physician.

[41] Collier SA, Deng L, Adam EA, Benedict KM, Beshearse EM, Blackstock AJ (2021). Estimate of burden and direct healthcare cost of infectious waterborne disease in the United States. Emerg Infect Dis.

[42.••] Crone S, Vives-Flórez M, Kvich L, Saunders AM, Malone M, Nicolaisen MH (2020). The environmental occurrence of Pseudomonas aeruginosa. APMIS.

[43] Januário AP, Afonso CN, Mendes S, Rodrigues MJ. Faecal indicator bacteria and pseudomonas aeruginosa in marine coastal waters: Is there a relationship? Pathogens. 2020;9. 10.3390/pathogens9010013. 10.3390/pathogens9010013 PMC7169392 31877730

[44] de Vicente A, Codina JC, Romero P (1991). Relationship between Pseudomonas aeruginosa and Bacterial Indicators in Polluted Natural Waters. Water Sci Technol.

[45] Liang L, Goh SG, Vergara GGRV, Fang HM, Rezaeinejad S, Chang SY (2015). Alternative fecal indicators and their empirical relationships with enteric viruses, Salmonella enterica, and Pseudomonas aeruginosa in surface waters of a tropical urban catchment. Appl Environ Microbiol.

[46] Santajit S, Indrawattana N. Mechanisms of Antimicrobial Resistance in ESKAPE Pathogens. Biomed Res Int. 2016;2016. 10.1155/2016/2475067. 10.1155/2016/2475067 PMC4871955 27274985

[47] Maragakis LL, Perl TM (2008). Acinetobacter baumannii: Epidemiology, antimicrobial resistance, and treatment options. Clin Infect Dis.

[48] Centers for Disease Control and Prevention (CDC). Antibiotic Resistance Threats in the United States, 2013. Atlanta, GA; 2013.

[49] Livermore DM (2002). Multiple mechanisms of antimicrobial resistance in Pseudomonas aeruginosa: Our worst nightmare?. Clin Infect Dis.

[50] Sekizuka T, Inamine Y, Segawa T, Hashino M, Yatsu K, Kuroda M (2019). Potential KPC-2 carbapenemase reservoir of environmental Aeromonas hydrophila and Aeromonas caviae isolates from the effluent of an urban wastewater treatment plant in Japan. Environ Microbiol Rep Wiley-Blackwell.

[51] Partridge SR, Kwong SM, Firth N, Jensen SO. Mobile genetic elements associated with antimicrobial resistance. Clin Microbiol Rev. 2018;31. 10.1128/CMR.00088-17. 10.1128/CMR.00088-17 PMC6148190 30068738

[52] Fournier PE, Vallenet D, Barbe V, Audic S, Ogata H, Poirel L (2006). Comparative genomics of multidrug resistance in Acinetobacter baumannii. PLoS Genet.

[53] Sakulworakan R, Chokmangmeepisarn P, Dinh-Hung N, Sivaramasamy E, Hirono I, Chuanchuen R, et al. Insight Into Whole Genome of Aeromonas veronii Isolated From Freshwater Fish by Resistome Analysis Reveal Extensively Antibiotic Resistant Traits. Front Microbiol. 2021;12. 10.3389/fmicb.2021.733668. 10.3389/fmicb.2021.733668 PMC8484913 34603262

[54] Do TT, Delaney S, Walsh F. 16S rRNA gene based bacterial community structure of wastewater treatment plant effluents. FEMS Microbiol Lett. 2019;366. 10.1093/femsle/fnz017. 10.1093/femsle/fnz017 30689818

[55] Carnelli A, Mauri F, Demarta A (2017). Characterization of genetic determinants involved in antibiotic resistance in Aeromonas spp. and fecal coliforms isolated from different aquatic environments. Res Microbiol..

[56] Seruga Music M, Hrenovic J, Goic-Barisic I, Hunjak B, Skoric D, Ivankovic T (2017). Emission of extensively-drug-resistant Acinetobacter baumannii from hospital settings to the natural environment. J Hosp Infect Elsevier Ltd.

[57] Higgins PG, Hrenovic J, Seifert H, Dekic S (2018). Characterization of Acinetobacter baumannii from water and sludge line of secondary wastewater treatment plant. Water Res Elsevier Ltd.

[58] Skwor T, Stringer S, Haggerty J, Johnson J, Duhr S, Johnson M, et al. Prevalence of potentially pathogenic antibiotic-resistant aeromonas spp. in treated urban wastewater effluents versus recipient riverine populations: A 3-year comparative study. Appl Environ Microbiol. 2020;86. 10.1128/AEM.02053-19. 10.1128/AEM.02053-19 PMC6974633 31757827

[59] Igbinosa IH, Nwodo UU, Sosa A, Tom M. Commensal Pseudomonas Species Isolated from Wastewater and Freshwater Milieus in the Eastern Cape Province , South Africa , as Reservoir of Antibiotic Resistant Determinants. 2012;2537–49. 10.3390/ijerph9072537. 10.3390/ijerph9072537 PMC3407919 22851958

[60] Slekovec C, Plantin J, Cholley P, Thouverez M, Talon D, Bertrand X, et al. Tracking Down Antibiotic-Resistant Pseudomonas aeruginosa Isolates in a Wastewater Network. PLoS One. 2012;7. 10.1371/journal.pone.0049300. 10.1371/journal.pone.0049300 PMC3526604 23284623

[61] Bosshard PP, Zbinden R, Abels S, Böddinghaus B, Altwegg M, Böttger EC (2006). 16S rRNA gene sequencing versus the API 20 NE system and the VITEK 2 ID-GNB card for identification of nonfermenting Gram-negative bacteria in the clinical laboratory. J Clin Microbiol.

[62] Wellinghausen N, Köthe J, Wirths B, Sigge A, Poppert S (2005). Superiority of molecular techniques for identification of gram-negative, oxidase-positive rods, including morphologically nontypical Pseudomonas aeruginosa, from patients with cystic fibrosis. J Clin Microbiol.

[63] Vanbroekhoven K, Ryngaert A, Wattiau P, de Mot R, Springael D (2004). Acinetobacter diversity in environmental samples assessed by 16S rRNA gene PCR-DGGE fingerprinting. FEMS Microbiol Ecol.

[64] Cherkaoui A, Emonet S, Renzi G, Schrenzel J. Characteristics of multidrug-resistant Acinetobacter baumannii strains isolated in Geneva during colonization or infection. Ann Clin Microbiol Antimicrob. 2015;14. 10.1186/s12941-015-0103-3. 10.1186/s12941-015-0103-3 PMC4567826 26361784

[65] Khan IUH, Loughborough A, Edge TA (2009). DNA-based real-time detection and quantification of aeromonads from fresh water beaches on Lake Ontario. J Water Health.

[66] Huddleston JR, Zak JC, Jeter RM (2006). Antimicrobial susceptibilities of Aeromonas spp. isolated from environmental sources. Appl Environ Microbiol..

[67] Cascón Soriano A, Anguita Castillo J, Hernanz Moral C, Sánchez Salazar M, Yugueros Marcos J, Naharro CG (1997). RFLP-PCR analysis of the aroA gene as a taxonomic tool for the genus Aeromonas. FEMS Microbiol Lett.

[68] Yousr AH, Napis S, Rusul GR, Son R (2007). Detection of Aerolysin and Hemolysin Genes in Aeromonas spp. Isolated from Environmental and Shellfish Sources by Polymerase Chain Reaction. ASEAN Food Journal..

[69] de Vos D, Lim A, Pirnay J-P, Struelens M, Vandenvelde C, Duinslaeger L (1997). Direct Detection and Identification of Pseudomonas aeruginosa in Clinical Samples Such as Skin Biopsy Specimens and Expectorations by Multiplex PCR Based on Two Outer Membrane Lipoprotein Genes, oprI and oprL. J Clin Microbiol.

[70] Lavenir R, Jocktane D, Laurent F, Nazaret S, Cournoyer B (2007). Improved reliability of Pseudomonas aeruginosa PCR detection by the use of the species-specific ecfX gene target. J Microbiol Methods.

[71] Qin X, Emerson J, Stapp J, Stapp L, Abe P, Burns JL (2003). Use of real-time PCR with multiple targets to identify Pseudomonas aeruginosa and other nonfermenting gram-negative bacilli from patients with cystic fibrosis. J Clin Microbiol.

[72] Khan AA, Cerniglia CE (1994). Detection of Pseudomonas aeruginosa from Clinical and Environmental Samples by Amplification of the Exotoxin A Gene Using PCR. Appl Environ Microbiol.

[73] Spilker T, Coenye T, Vandamme P, LiPuma JJ (2004). PCR-Based Assay for Differentiation of Pseudomonas aeruginosa from Other Pseudomonas Species Recovered from Cystic Fibrosis Patients. J Clin Microbiol.

[74] Schwartz T, Volkmann H, Kirchen S, Kohnen W, Schön-Hölz K, Jansen B (2006). Real-time PCR detection of Pseudomonas aeruginosa in clinical and municipal wastewater and genotyping of the ciprofloxacin-resistant isolates. FEMS Microbiol Ecol.

[75] Lee CS, Wetzel K, Buckley T, Wozniak D, Lee J (2011). Rapid and sensitive detection of Pseudomonas aeruginosa in chlorinated water and aerosols targeting gyrB gene using real-time PCR. J Appl Microbiol.

[76] Beaz-Hidalgo R, Hossain MJ, Liles MR, Figueras MJ. Strategies to avoid wrongly labelled genomes using as example the detected wrong taxonomic affiliation for aeromonas genomes in the genbank database. PLoS One. 2015;10. 10.1371/journal.pone.0115813. 10.1371/journal.pone.0115813 PMC4301921 25607802

[77] Po-Lin C, Chi-Jung W, Pei-Jane T, Hung-Jen T, Yin-Ching C, Nan-Yao L, et al. Virulence diversity among bacteremic aeromonas isolates: Ex Vivo, animal, and clinical evidences. PLoS One. Public Library of Science; 2014;9. 10.1371/journal.pone.0111213. 10.1371/journal.pone.0111213 PMC4222899 25375798

[78] Radó J, Kaszab E, Benedek T, Kriszt B, Szoboszlay S (2019). First isolation of carbapenem-resistant Acinetobacter beijerinckii from an environmental sample. Acta Microbiol Immunol Hung.

[79] Singhal N, Kumar M, Kanaujia PK, Virdi JS. MALDI-TOF mass spectrometry: An emerging technology for microbial identification and diagnosis. Front Microbiol. 2015;6. 10.3389/fmicb.2015.00791. 10.3389/fmicb.2015.00791 PMC4525378 26300860

[80] Tran A, Alby K, Kerr A, Jones M, Gilligan PH (2015). Cost savings realized by implementation of routine microbiological identification by matrix-assisted laser desorption ionization-time of flight mass spectrometry. J Clin Microbiol.

[81] Chen PL, Lee TF, Wu CJ, Teng SH, Teng LJ, Ko WC (2014). Matrix-assisted laser desorption ionization-time of flight mass spectrometry can accurately differentiate Aeromonas dhakensis from A hydrophila, A. caviae, and A. veronii. J Clin Microbiol..

[82] Chen PL, Lamy B, Ko WC. Aeromonas dhakensis, an increasingly recognized human pathogen. Front Microbiol. 2016;7. 10.3389/fmicb.2016.00793. 10.3389/fmicb.2016.00793 PMC4882333 27303382

[83] Marí-Almirall M, Cosgaya C, Higgins PG, van Assche A, Telli M, Huys G, et al. MALDI-TOF/MS identification of species from the Acinetobacter baumannii (Ab) group revisited: inclusion of the novel A. seifertii and A. dijkshoorniae species. Clin Microbiol Infect. 2017;23. 10.1016/j.cmi.2016.11.020. 10.1016/j.cmi.2016.11.020 27919649

[84] Li J, Hu W, Li M, Deng S, Huang Q, Lu W (2019). Evaluation of matrix-assisted laser desorption/ ionization time-of-flight mass spectrometry for identifying VIM-and SPM-type metallo-β-lactamase-producing pseudomonas aeruginosa clinical isolates. Infect Drug Resist.

[85] Chaix G, Roger F, Berthe T, Lamy B, Jumas-Bilak E, Lafite R, et al. Distinct Aeromonas populations in water column and associated with copepods from estuarine environment (Seine, France). Front Microbiol. 2017;8. 10.3389/fmicb.2017.01259. 10.3389/fmicb.2017.01259 PMC5504101 28744262

[86] Verburg I, García-Cobos S, Leal LH, Waar K, Friedrich AW, Schmitt H. Abundance and antimicrobial resistance of three bacterial species along a complete wastewater pathway. Microorganisms. 2019;7. 10.3390/microorganisms7090312. 10.3390/microorganisms7090312 PMC6780886 31484380

[87] Rhodes G, Huys G, Swings J, McGann P, Hiney M, Smith P (2000). Distribution of oxytetracycline resistance plasmids between aeromonads in hospital and aquaculture environments: Implication of Tn1721 in dissemination of the tetracycline resistance determinant Tet A. Appl Environ Microbiol.

[88] Huddleston JR, Zak JC, Jeter RM (2007). Sampling bias created by ampicillin in isolation media for Aeromonas. Can J Microbiol.

[89] Suzuki Y, Kajii S, Nishiyama M, Iguchi A (2013). Susceptibility of Pseudomonas aeruginosa isolates collected from river water in Japan to antipseudomonal agents. Sci Total Environ.

[90] Santoro DO, Cardoso AM, Coutinho FH, Pinto LH, Vieira RP, Albano RM (2015). Diversity and antibiotic resistance profiles of Pseudomonads from a hospital wastewater treatment plant. J Appl Microbiol.

[91] Igbinosa EO, Odjadjare EE, Igbinosa IH, Orhue PO, Omoigberale MNO, Amhanre NI. Antibiotic synergy interaction against multidrug-resistant Pseudomonas aeruginosa isolated from an abattoir effluent environment. Sci World J. 2012;2012. 10.1100/2012/308034. 10.1100/2012/308034 PMC3353294 22629128

[92] King EO, Ward MK, Raney DE (1954). Two simple media for the demonstration of pyocyanin and fluorescin. J Lab Clin Med.

[93] Lowbury EJL, Collins AG (1955). The use of a new cetrimide product in a selective medium for Pseudomonas pyocyanea. J Clin Pathol.

[94] Lowbury EJL (1951). Contamination of cetrimide and other fluids with Pseudomonas pyocyanea. Br J Ind Med.

[95] International Organization for Standardization (2018). Water quality — Detection and enumeration of Pseudomonas aeruginosa — Method by membrane filtration (ISO 16266–2:2018).

[96] Baird R, Eaton A, Rice E, editors. Method 9213 Recreational Waters E-F. Standard methods for the examination of water and wastewater. 21st ed. Washington, D.C.: APHA-AWWA-WEF; 2005.

[97] United States Environmental Protection Agency (U.S. EPA). Method 1605: Aeromonas in Finished Water by Membrane Filtration using Ampicillin-Dextrin Agar with Vancomycin (ADA-V) (October 2001). 2001;EPA-821-R-01–034.

[98] Skwor T, Shinko J, Augustyniak A, Gee C, Andraso G (2014). Aeromonas hydrophila and Aeromonas veronii predominate among potentially pathogenic ciprofloxacin- and tetracycline-resistant Aeromonas isolates from Lake Erie. Appl Environ Microbiol.

[99] Lilly HA, Lowbury EJL (1972). Cetrimide-nalidixic acid as a selective medium for Pseudomonas aeruginosa. J Med Microbiol.

[100] Kodaka H, Iwata M, Yumoto S, Kashitani F (2003). Evaluation of a new agar medium containing cetrimide, kanamycin and nalidixic acid for isolation and enhancement of pigment production of Pseudomonas aeruginosa in clinical samples. J Basic Microbiol.

[101] Zhang Z, Zhang Q, Wang T, Xu N, Lu T, Hong W, et al. Assessment of global health risk of antibiotic resistance genes. Nat Commun. 2022;13. 10.1038/s41467-022-29283-8. 10.1038/s41467-022-29283-8 PMC8943045 35322038

[102] Li H, Liu F, Zhang Y, Wang X, Zhao C, Chen H (2015). Evolution of carbapenem-resistant Acinetobacter baumannii revealed through whole-genome sequencing and comparative genomic analysis. Antimicrob Agents Chemother.

[103] Turton JF, Ward ME, Woodford N, Kaufmann ME, Pike R, Livermore DM (2006). The role of ISAba1 in expression of OXA carbapenemase genes in Acinetobacter baumannii. FEMS Microbiol Lett.

[104] Anane AY, Apalata T, Vasaikar S, Okuthe GE, Songca S (2019). Prevalence and molecular analysis of multidrug-resistant Acinetobacter baumannii in the extra-hospital environment in Mthatha, South Africa. Braz J Infect Dis.

[105] Goic-Barisic I, Seruga Music M, Kovacic A, Tonkic M, Hrenovic J (2017). Pan Drug-Resistant Environmental Isolate of Acinetobacter baumannii from Croatia. Microb Drug Resist.

[106] Goic-Barisic I, Hrenovic J, Kovacic A, Musić MŠ (2016). Emergence of Oxacillinases in Environmental Carbapenem-Resistant Acinetobacter baumannii Associated with Clinical Isolates. Microb Drug Resist.

[107] Kovacic A, Music MS, Dekic S, Tonkic M, Novak A, Rubic Z (2017). Transmission and survival of carbapenem-resistant Acinetobacter baumannii outside hospital setting. Int Microbiol.

[108] Igbinosa IH, Okoh AI. Antibiotic susceptibility profile of aeromonas species isolated from wastewater treatment plant. Sci World J. 2012;2012. 10.1100/2012/764563. 10.1100/2012/764563 PMC3425809 22927788

[109] Moura A, Pereira C, Henriques I, Correia A (2012). Novel gene cassettes and integrons in antibiotic-resistant bacteria isolated from urban wastewaters. Res Microbiol.

[110] Piotrowska M, Przygodzinska D, Matyjewicz K, Popowska M. Occurrence and variety of ß-lactamase genes among Aeromonas spp. isolated from Urban Wastewater treatment plant. Front Microbiol. 2017;8. 10.3389/fmicb.2017.00863. 10.3389/fmicb.2017.00863 PMC5432545 28559885

[111] Camiade M, Bodilis J, Chaftar N, Riah-Anglet W, Gardères J, Buquet S (2020). Antibiotic resistance patterns of Pseudomonas spp. isolated from faecal wastes in the environment and contaminated surface water. FEMS Microbiol Ecol..

[112] Chikwendu CI, Ibe SN, Okpokwasili GC (2011). Detection of bla SHV and bla TEM beta-lactamase genes in multi-resistant Pseudomonas isolates from environmental sources.

[113] Olga P, Apostolos V, Alexis G, George V, Athena M. Antibiotic resistance profiles of Pseudomonas aeruginosa isolated from various Greek aquatic environments. FEMS Microbiol Ecol. 2016;92. 10.1093/femsec/fiw042. 10.1093/femsec/fiw042 26917780

[114] Oliveira LG, Ferreira LGR, Nascimento AMA, Reis MDP, Dias MF, Lima WG (2017). Antibiotic resistance profile and occurrence of Amp C between Pseudomonas aeruginosa isolated from a domestic full-scale WWTP in southeast Brazil. Water Sci Technol.

[115] Zanetti MO, Martins VV, Pitondo-Silva A, Stehling EG (2013). Antimicrobial resistance, plasmids and class 1 and 2 integrons occurring in Pseudomonas aeruginosa isolated from Brazilian aquatic environments. Water Sci Technol.

[116] Buehrle DJ, Wagener MM, Nguyen MH, Clancy CJ. Trends in Outpatient Antibiotic Prescriptions in the United States during the COVID-19 Pandemic in 2020. JAMA Netw Open. 2021;4. 10.1001/jamanetworkopen.2021.26114. 10.1001/jamanetworkopen.2021.26114 PMC8459187 34550387

[117] Krause KM, Serio AW, Kane TR, Connolly LE. Aminoglycosides: An overview. Cold Spring Harb Perspect Med. 2016;6. 10.1101/cshperspect.a027029. 10.1101/cshperspect.a027029 PMC4888811 27252397

[118] Kanafani ZA, Kanj SS. Acinetobacter infection: Treatment and prevention. In: Calderwood SB, Hall KK, editors. UpToDate. Waltham, MA: UpToDate Inc.; 2022. Available from: https://www.uptodate.com. Accessed 14 Apr 2022.

[119] World Health Organization (WHO). AWaRe [Internet]. 2019. Available from: https://adoptaware.org/. Accessed 3 May 2021.

[120] Chen PL, Ko WC, Wu CJ (2012). Complexity of β-lactamases among clinical Aeromonas isolates and its clinical implications. J Microbiol Immunol Infect.

[121] Wu CJ, Chen PL, Hsueh PR, Chang MC, Tsai PJ, Shih HI, et al. Clinical implications of species identification in monomicrobial Aeromonas bacteremia. PLoS One. 2015;10. 10.1371/journal.pone.0117821. 10.1371/journal.pone.0117821 PMC4334500 25679227

[122] de Luca F, Giraud-Morin C, Rossolini GM, Docquier JD, Fosse T (2010). Genetic and biochemical characterization of TRU-1, the endogenous class C β-lactamase from Aeromonas enteropelogenes. Antimicrob Agents Chemother.

[123] Magiorakos AP, Srinivasan A, Carey RB, Carmeli Y, Falagas ME, Giske CG (2012). Multidrug-resistant, extensively drug-resistant and pandrug-resistant bacteria: An international expert proposal for interim standard definitions for acquired resistance. Clin Microbiol Infect..

[124] Harnisz M, Tucholski S (2010). Microbial quality of common carp and pikeperch fingerlings cultured in a pond fed with treated wastewater. Ecol Eng..

[125] Santoro DO, Clementino MM (2012). Decreased aztreonam susceptibility among Pseudomonas aeruginosa isolates from hospital effluent treatment system and clinical samples.

[126] Wu CJ, Chen PL, Wu JJ, Yan JJ, Lee CC, Lee HC (2012). Distribution and phenotypic and genotypic detection of a metallo-β-lactamase, CphA, among bacteraemic Aeromonas isolates. J Med Microbiol.

[127] Wu CJ, Wang HC, Chen PL, Chang MC, Sunny Sun H, Chou PH (2013). AQU-1, a chromosomal class C β-lactamase, among clinical Aeromonas dhakensis isolates: Distribution and clinical significance. Int J Antimicrob Agents.

[128] Benagli C, Demarta A, Caminada AP, Ziegler D, Petrini O, Tonolla M (2012). A Rapid MALDI-TOF MS Identification Database at Genospecies Level for Clinical and Environmental Aeromonas Strains. PLoS ONE.

[129.••] Benoit T, Cloutier M, Schop R, Lowerison MW, Khan IUH. Comparative assessment of growth media and incubation conditions for enhanced recovery and isolation of Acinetobacter baumannii from aquatic matrices. J Microbiol Methods. Elsevier; 2020;176:106023. **Only study found evaluating CHROMagar Acinetobacter for use on a variety of aquatic environmental samples under different incubation conditions and a direct comparison to another selective medium frequently used for clinical samples.** 10.1016/j.mimet.2020.106023 32795636

[130] International Organization for Standardization (2005). Water quality — General guidance on the enumeration of micro-organisms by culture (ISO 8199: 2005).

[131] Liguori K, Keenum I, Davis B, Milligan E, Heath LS, Pruden A (2023). Standardizing methods with QA/QC standards for investigating the occurrence and removal of Antibiotic Resistant Bacteria/Antibiotic Resistance Genes (ARB/ARGs) in surface water, wastewater, and recycled water. Project 5052.

