# Supplementary material for: A Systematic Review of Culture-Based Methods for Monitoring Antibiotic-Resistant Acinetobacter, Aeromonas, and Pseudomonas as Environmentally Relevant Pathogens in Wastewater and Surface Water
Source: Curr Environ Health Rep. 2023 Feb 23;10(2):154–71. doi: 10.1007/s40572-023-00393-9 (PMC10299953; doi:10.1007/s40572-023-00393-9)
Supplement: Supplementary file 1 — Supplementary file1 (DOCX 235 kb) [file 40572_2023_393_MOESM1_ESM.docx]

**Supplementary Material**

**Containing Figures S1-S3 and Tables S1 & S2**

**A systematic review of culture-based methods for monitoring antibiotic resistant *Acinetobacter*, *Aeromonas*, and *Pseudomonas* as environmentally-relevant pathogens in wastewater and surface water**

Authors: Erin G. Milligan^1,2^, Jeanette Calarco^3^, Benjamin C. Davis^1^, PhD, Ishi M. Keenum^1^, PhD, Krista Liguori^1^, Amy Pruden^1,2*^, PhD, Valerie J. Harwood^3*^, PhD

^1^ Via Department of Civil and Environmental Engineering, Virginia Tech, Blacksburg, Virginia

^2^ Center for Emerging, Zoonotic, and Arthropod-Borne Pathogens, Virginia Polytechnic Institute and State University, Blacksburg, VA 24061, USA.

^3^ Department of Integrative Biology, University of South Florida, Tampa, Florida

* Co-corresponding authors Harwood (Department of Integrative Biology, University of South Florida, Tampa, FL 33620, USA, [vharwood@usf.edu](mailto:vharwood@usf.edu)) and Pruden (Department of Civil and Environmental Engineering, Virginia Tech, Blacksburg, Virginia 24060, USA, [apruden@vt.edu](mailto:apruden@vt.edu))

Web of Science and PubMed records identified after removal of duplicates (n = 810)

**Identification**

Records excluded

(n = 627)

Titles/Abstracts screened

(n = 810)

**Screening**

Studies excluded:

- Used non-selective isolation media or an enrichment step (n = 80)
- Culture methods not described (n = 22)
- Irrelevant sample type

(n = 18)

- No antibiotic resistance assessment of isolates

(n = 3)

Full-text articles assessed for eligibility (n = 183)

Studies included in review (n = 60)

**Included**

**Fig. S1** Overview of PRISMA strategy used for selection of articles to include in this systematic review

**Table S1** Systematic review search terms

| **Tier** | **Search Terms** |
| --- | --- |
| 1 | TS = ( “antibiotic resistan*” OR “antimicrobial susceptibility” OR “antimicrobial resistan*” OR “drug resistan*” OR “multi-drug resistan*” OR “resistome” OR ”ARG” OR “antibiotic resistan* gene”) |
| 2 | TS = (“wastewater” OR “reclaimed water” OR “recycled water” OR “water reuse” OR “non-potable reuse” OR “greywater” OR “hospital wastewater” OR “surface water” OR “sewage” OR “wastewater treatment plant” OR “filtration” OR “direct potable reuse” OR “indirect potable reuse” OR “river” OR “watershed” OR “lake” OR “pond” OR “recreational water” OR “influent” OR “effluent” OR “aquatic” OR “water quality” OR “de facto reuse” ) |
| 3 | TS =(“culture” OR “dis* diffusion” OR “isolat*” OR "membrane filtrat*" OR "spread plating" OR "IDEXX" OR "Colilert" OR "Colilert-18" OR "Colisure" OR "Enterolert" OR "Pseudalert" OR "Enterolert-E") |
| 4 | (4) TS =(“Acinetobacter” OR “A. baumannii” OR “Aeromonas” OR “Pseudomonas” OR “P. aeruginosa”) |

**Table S2** Detailed information on selective isolation media

| ***Acinetobacter* spp.** | | | |
| --- | --- | --- | --- |
| **Media** | **Typical Ingredients** | **Color-based differential? (Y/N)** | **Reference or Supplier** |
| CHROMagar Acinetobacter | Peptone and yeast extract, salts, Chromogenic mix, agar | Y  *Acinetobacter* spp. (red) | CHROMagar^TM^ |
| Acinetobacter broth/ Baumann agar | Sodium acetate (trihydrate), potassium nitrate, magnesium sulfate, monopotassium/monosodium phosphate buffer, Hutner’s mineral base | N | [1] |
| Leeds Acinetobacter (LAM) | Acid casein hydrolysate, neutralized soy peptone, sodium chloride, D-(-)-fructose, sucrose, D-mannitol, L-phenylalanine, ferric ammonium citrate, phenol red, vancomycin, cefsulodin, cephradine, agar | Y  *Acinetobacter* spp. (mauve) | [2] |
| ***Aeromonas* spp.** | | | |
| **Media** | **Typical Ingredients** | **Color-based differential? (Y/N)** | **Reference or Supplier** |
| Glutamate Starch Phenol Red (GSP) | L-glutamate, soluble starch, monopotassium phosphate, magnesium sulfate, phenol red, agar | Y  *Aeromonas* (yellow)  *Pseudomonas*  (blue-violet) | [3] |
| Ampicillin-dextrin (ADA) | Ampicillin sodium salt, tryptose, dextrin, yeast extract, sodium chloride, potassium chloride, magnesium sulfate heptahydrate, ferric chloride hexahydrate, bromothymol blue, sodium deoxycholate, agar | Y  *Aeromonas* spp. (yellow) | [4] |
| Ryan’s Aeromonas (RYAN) | Ampicillin, proteose peptone, yeast extract, L-lysine monohydrochloride, L-arginine monohydrochloride, sorbitol, inositol, lactose, xylose, bile salts, sodium thiophosphate, sodium chloride, ferric ammonium citrate, bromothymol blue, thymol blue, agar | Y  *Aeromonas* (dark green-opaque)  *Pseudomonas* (blue-gray translucent) | Oxoid^TM^  Aeromonas Medium Base (RYAN) |
| Aeromonas Isolation (AIA) | Soluble starch, NH4Cl, tryptone, yeast extract, bromothymol blue, agar, sodium deoxycholate, L-tryptophan, L-phenylalanine | N | [5] |
| Rimler-Shotts | Yeast extract, maltose, L-cysteine hydrochloride, L-lysine hydrochloride, sodium thiosulphate, ferric ammonium citrate, sodium deoxycholate, sodium chloride, bromothymol blue, agar | Y  *Aero. hydrophila* (yellow) | [6] |
| Aero-Smart AH | L-lysine, l-orthinine, maltose, sodium thiosulphate, L-cysteine, bromothymol blue, ferric ammonium citrate, bile salts, NaCl, yeast extract, agar | Y  *Aeromonas* spp.  (yellow) | [7] |
| Ampicillin-trehalose (mA) | Ampicillin, sodium deoxycholate, tryptose, trehalose, yeast extract, sodium chloride, potassium chloride, magnesium sulfate heptahydrate, ferric chloride hexahydrate, bromothymol blue, agar | Y  *Aero. hydrophila* (yellow) | [8] |
| Pril-ampicillin-dextrin-ethanol (PADE) | Ampicillin, sodium deoxycholate, ethanol, tryptose, yeast extract, dextrin Weiss, pril, magnesium sulfate heptahydrate, ferric chloride hexahydrate, bromothymol blue, thymol blue, agar | Y  *Aeromonas* spp. (yellow) | [9] |
| Xylose deoxycholate citrate (XDCA) | Xylose, neutral red, nutrient broth, sodium citrate, sodium thiosulphate, ferric ammonium citrate, sodium deoxycholate, agar | Y  *Aeromonas* spp. (colorless) | [10,11] |
| ***Pseudomonas* spp.** | | | |
| **Media** | **Typical Ingredients** | **Color-based differential? (Y/N)** | **Reference or Supplier** |
| Cetrimide | Gelatin peptone, magnesium chloride, potassium sulphate, cetrimide, agar | Y  *P. aeruginosa* (green) | [12,13] |
| Cetrimide-nalidixic-acid (Pseudomonas CN) | Gelatin peptone, casein hydrolysate, magnesium chloride, potassium sulphate, cetrimide, nalidixic acid, agar | Y  *P. aeruginosa* (green) | [14] |
| Pseudomonas Isolation (PIA) | Gelatin peptone, potassium sulfate, magnesium chloride, irgasan, glycerol, agar | Y  *P. aeruginosa* (green) | [15] |
| GSP | L-glutamate, soluble starch, monopotassium phosphate, magnesium sulfate, phenol red, agar | Y  *Aeromonas* (yellow)  *Pseudomonas*  (blue-violet) | [3] |
| Cetrimide-nalidixic-acid-kanamycin (CKNA) | Gelatin peptone, magnesium chloride, potassium sulphate, cetrimide, nalidixic acid, kanamycin, agar | Y  *P. aeruginosa* (green) | [16] |
| Cetrimide broth | Peptic digest of animal tissue, beef extract, sodium chloride, cetrimide | Y  *P. aeruginosa* (green fluorescent) | [15] |
| Asparagine broth | Dipotassium phosphate, DL-asparagine, magnesium sulfate | Y  *P. aeruginosa* (green fluorescent) | [17] |
| M-PA-C | Yeast extract, L-lysine HCl, sodium chloride, xylose, sucrose, lactose, phenol red, ferric ammonium citrate, sodium thiosulfate, magnesium sulfate, kanamycin, nalidixic acid, agar | Y  *P. aeruginosa* (dark brown or green-black centers) | [18] |
| Cephalosporin-fucidin-cetrimide (CFC) | Gelatin peptone, casein hydrolysate, magnesium chloride, potassium sulphate, cephalothin, sodium fusidate, cetrimide | N | [19] |
| Fluorescein denitrification (FNA) | Peptic digest of animal tissue, casein hydrolysate, potassium nitrate, magnesium sulfate, dipotassium phosphate, agar | Y  *Pseudomonas* spp. (fluorescent) | [20] |
| Pseudomonas denitrificans | Glucose, yeast extract, ferric chloride agar | N | [20] |
| Malachite green broth | Peptone from meat, meat extract, di-potassium hydrogen phosphate, malachite green oxalate | N | [21] |


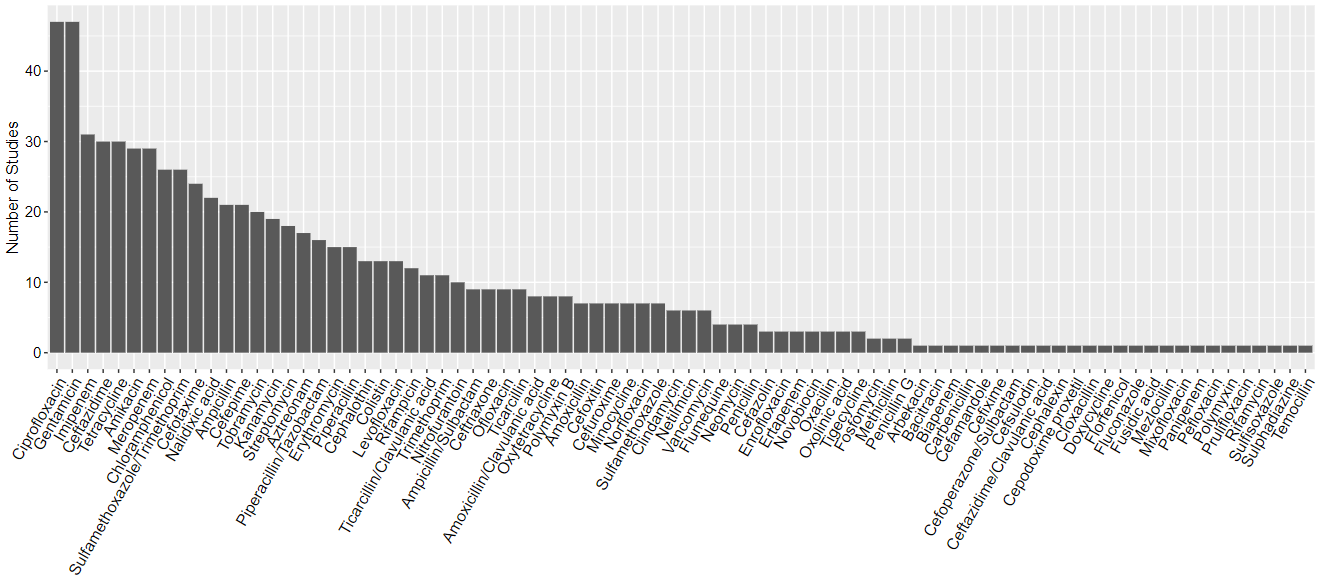


**Fig. S2** Frequency of all antibiotics tested for phenotypic resistance among isolates (*Acinetobacter* spp., *Aeromonas* spp., and *Pseudomonas* spp.) across studies.

*
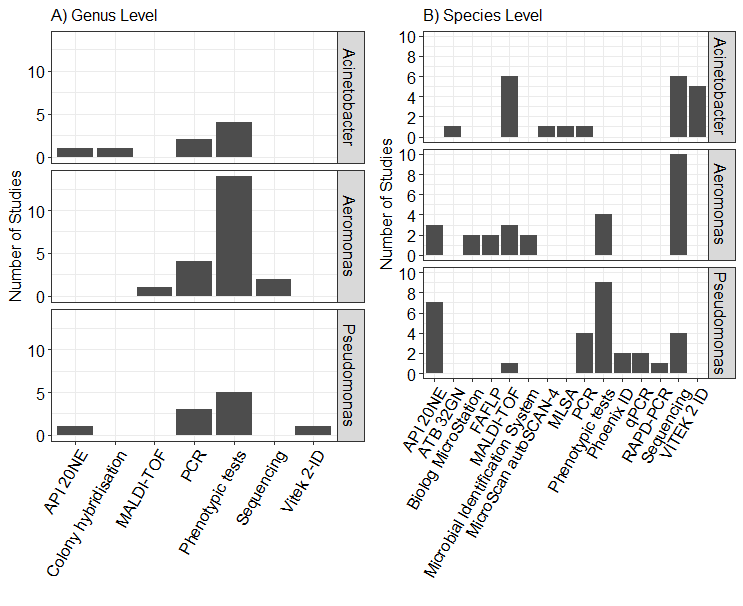
*

**Fig. S3** Frequently reported methods for isolate confirmation at the genus level (panel A, n = 32 studies) and species level (panel B, n = 48 studies).

References

1. Baumann P. Isolation of Acinetobacter from soil and water. J Bacteriol. 1968;96:39–42.

2. Jawad A, Hawkey PM, Heritage J, Snelling AM. Description of Leeds Acinetobacter Medium, a New Selective and Differential Medium for Isolation of Clinically Important Acinetobacter spp., and Comparison with Herellea Agar and Holton’s Agar. J Clin Microbiol. 1994.

3. Kielwein G. Pseudomonaden und Aromonaden in Trinkmilch: Ihr Nachweis und ihre Bewertung. Arch f Lebensmittelhyg. 1971;22:15–9.

4. United States Environmental Protection Agency (U.S. EPA). Method 1605: Aeromonas in Finished Water by Membrane Filtration using Ampicillin-Dextrin Agar with Vancomycin (ADA-V) (October 2001). 2001.

5. Huddleston JR, Zak JC, Jeter RM. Antimicrobial susceptibilities of Aeromonas spp. isolated from environmental sources. Appl Environ Microbiol. 2006;72:7036–42.

6. Shotts EB, Rimler R. Medium for the Isolation of Aeromonas hydrophila. Appl Microbiol. 1973;26:550–3.

7. Odeyemi OA, Ahmad A. Population dynamics, antibiotics resistance and biofilm formation of Aeromonas and Vibrio species isolated from aquatic sources in Northern Malaysia. Microb Pathog. Elsevier Ltd; 2017;103:178–85.

8. Rippey SR, Cabelli VJ. Membrane filter procedure for enumeration of Aeromonas hydrophila in fresh waters. Appl Environ Microbiol. 1979;38:108–13.

9. Imziln B, Lafdal OMY, Barakate M, Hassani L, Ouhdouch Y, Boussaid A, et al. Pril-ampicillin-dextrin-ethanol agar for the isolation and quantification of Aeromonas spp. From polluted environmental waters. J Appl Microbiol. Blackwell Publishing Ltd; 1997;82:557–66.

10. Millership SE, Chattopadhyay B. Methods for the isolation of Aeromonas hydrophila and Plesiomonas shigelloides from faeces. Journal of Hygiene. 1984;92:145–52.

11. Shread P, Donovan TJ, Lee J v. A survey of the incidence of Aeromonas in human faeces. Society for General Microbiology Quarterly. 1981;8:184.

12. Lowbury EJL. Contamination of cetrimide and other fluids with Pseudomonas pyocyanea. Br J Ind Med. 1951;8:22–5.

13. Lowbury EJ, Collins AG. The use of a new cetrimide product in a selective medium for Pseudomonas pyocyanea. J Clin Pathol. 1955;8:47–8.

14. Lilly HA, Lowbury EJL. Cetrimide-nalidixic acid as a selective medium for Pseudomonas aeruginosa. J Med Microbiol. 1972;5:151–3.

15. King EO, Ward MK, Raney DE. Two simple media for the demonstration of pyocyanin and fluorescin. J Lab Clin Med. United States; 1954;44:301–7.

16. Kodaka H, Iwata M, Yumoto S, Kashitani F. Evaluation of a new agar medium containing cetrimide, kanamycin and nalidixic acid  for isolation and enhancement of pigment production of Pseudomonas aeruginosa in clinical samples. J Basic Microbiol. Germany; 2003;43:407–13.

17. Clersceri LS, Greenburg AE, Eaton AD. Standard methods for the examination of water and wastewater. 20th ed. 1998.

18. Dutka BJ, Kwan KK. Confirmation of the single step membrane filtration procedure for estimating Pseudomonas aeruginosa densities in water. Appl Environ Microbiol. 1977;33:240–5.

19. Mead GC, Adams BW. A selective medium for the rapid isolation of pseudomonads associated with poultry meat spoilage. Br Poult Sci [Internet]. Taylor & Francis; 1977;18:661–70. Available from: https://doi.org/10.1080/00071667708416418

20. Atlas RM. Handbook of microbiological media. Boca Raton, Fla.; 2010.

21. HABS, H., a. KIRSCHNER, K.H.: Der Pyocyaneus-Meerschweinchenhautversuch zur Prüfung von Hautdesinfektionsmitteln. – Z. Hyg., 1943;124:557-578
